# HbAHP-25, an *In-Silico* Designed Peptide, Inhibits HIV-1 Entry by Blocking gp120 Binding to CD4 Receptor

**DOI:** 10.1371/journal.pone.0124839

**Published:** 2015-04-27

**Authors:** Tahir Bashir, Mandar Patgaonkar, Selvaa Kumar C, Achhelal Pasi, Kudumula Venkata Rami Reddy

**Affiliations:** 1 Division of Molecular Immunology and Microbiology, National Institute for Research in Reproductive Health, Indian Council of Medical Research, Mumbai, India; 2 Department of Biological Sciences, Tata Institute for Fundamental Research, Mumbai, India; 3 Department of Bioinformatics, School of Biotechnology and Bioinformatics, D.Y. Patil University, Navi Mumbai, India; Rutgers University, UNITED STATES

## Abstract

Human Immunodeficiency Virus (HIV-1) poses a serious threat to the developing world and sexual transmission continues to be the major source of new infections. Therefore, the development of molecules, which prevent new HIV-1 infections, is highly warranted. In the present study, a panel of human hemoglobin (Hb)-α subunit derived peptides and their analogues, with an ability to bind gp120, were designed *in-silico* and their anti-HIV-1 activity was evaluated. Of these peptides, HbAHP-25, an analogue of Hb-α derived peptide, demonstrated significant anti-HIV-1 activity. HbAHP-25 was found to be active against CCR5-tropic HIV-1 strains (ADA5 and BaL) and CXCR4-tropic HIV-1 strains (IIIB and NL4-3). Surface plasmon resonance (SPR) and ELISA revealed direct interaction between HbAHP-25 and HIV-1 envelope protein, gp120. The peptide prevented binding of CD4 to gp120 and blocked subsequent steps leading to entry and/or fusion or both. Anti-HIV activity of HbAHP-25 appeared to be specific as it failed to inhibit the entry of HIV-1 pseudotyped virus (HIV-1 VSV). Further, HbAHP-25 was found to be non-cytotoxic to TZM-bl cells, VK2/E6E7 cells, CEM-GFP cells and PBMCs, even at higher concentrations. Moreover, HbAHP-25 retained its anti-HIV activity in presence of seminal plasma and vaginal fluid. In brief, the study identified HbAHP-25, a novel anti-HIV peptide, which directly interacts with gp120 and thus has a potential to inhibit early stages of HIV-1 infection.

## Introduction

AIDS (Acquired Immunodeficiency Syndrome), caused by Human Immunodeficiency Virus (HIV), is one of the leading causes of death worldwide [1]. Statistics reveal that in 2012 alone, approximately 1.7 million deaths were caused by AIDS, and 2.5 million people were newly infected by the virus [2]. Currently 34 million people are living with HIV worldwide, and 8 million are on anti-retrovirals [2]. Although various antiretroviral drugs have been found efficacious as anti-HIV therapeutics, strategies focused on the prevention of new infections are expected to have far reaching implications in terms of reducing the burden on health care system worldwide.

HIV-1 infection can be targeted at various stages, for example, viral entry, viral replication or assembly of viral components. HIV entry into the host cells is facilitated by binding of viral envelope glycoprotein (gp120) to host CD4 receptor [3, 4]. CD4-gp120 interaction initiates a cascade of events that stimulates gp41 to promote viral and host membrane fusion [4]. Inhibition of gp120-CD4 interaction or virus-host cell fusion thus appears to be an attractive strategy to prevent HIV-1 infection. Towards this, significant advances have been made. Enfuviritide (T-20), the first drug approved for clinical use by food and drug administration (FDA), has been shown to alleviate HIV infections effectively [5, 6]. Several low-molecular-weight (LMW) compounds and antimicrobial peptides (AMPs), which interfere with the initial steps of virus entry either by targeting gp120, gp41 or CCR5/CXCR4 co-receptors, have also been discovered [7–14]. However, their efficacy in clinical settings remains to be established.

For the past few years, our research has been directed towards the development of AMPs with anti-HIV activity. We previously demonstrated the presence of hemoglobin derived antimicrobial peptides in rabbit vaginal fluid. One of these peptides, rabbit vaginal fluid hemoglobin alpha-peptide (RVFHbαP), showed 96% sequence homology with that of human hemoglobin-α (Hb-α) subunit [15]. This peptide was found to be active against several Gram-positive and Gram-negative bacteria. Various reports have shown that hemoglobin (Hb) derived peptides (hemocidins) act as first line of host defense against several invading pathogens [16]. Some hemocidins were found to have antimicrobial activity comparable to that of defensins, cathelicidins etc. [17]. Hb derived peptides have been shown to prevent microbial infections during menstruation [18, 19]. However, till date the anti-HIV potential of hemoglobin derived antimicrobial peptides has not been explored. This prompted us to investigate whether Hb derived peptides have anti-HIV activity. We were also encouraged by the fact that hemoglobin (Hb) is an essential and abundant protein in humans and hemocidins naturally exist in human vagina. Hence, adverse immunologic effects are not expected in response to Hb-derived peptides.

The present study was undertaken: 1) to design Hb-α subunit derived peptides with an ability to bind gp120; 2) to assess the anti-HIV activity of Hb-derived peptides using different assays and 3) to decipher the mechanism by which these peptides exert anti-HIV activity. Here, we report that human Hb-α derived peptide analogue, HbAHP-25 (hemoglobin anti-HIV peptide-25), has substantial anti-HIV activity. HbAHP-25 binds to CD4 binding region of gp120 of HIV-1 and thereby interferes with gp120-CD4 interaction. This peptide did not show any adverse effect on viability of cells, even at a dose 3-fold higher than its IC_50_. Further, the activity of HbAHP-25 was found to be specific as it did not inhibit the entry of HIV-1 pseudotyped virus (HIV-1 VSV). In addition, HbAHP-25 retained its anti-HIV activity in the presence of human seminal plasma and vaginal fluid. To the best of our knowledge, this is the first report on an Hb-derived AMP with anti-HIV activity.

## Materials and Methods

### Animals

Sexually mature female rats (Holtzman strain) (body weight, 190 ± 25 gm) were maintained under standard housing conditions (temperature 20±1°C, relative humidity 50 ± 10% and 12h light: 12h darkness cycle) and immunized with peptide according to the protocol approved by the Institutional Ethics Committee (NIRRH/IAEC: 1/07) NIRRH, Mumbai. Handling of rats was according to the guidelines for care and use of laboratory animals.

### Ethics Statement

The study design was approved for the use of human biological samples (blood/semen/vaginal lavage) by the Institutional Ethics Committee for Clinical Studies (D/ICEC/Sci-13/18/2014). Written informed consent was obtained from individuals prior to their participation. Samples were collected from normal, healthy adult volunteers (age 21–40 years).

### Culture Media and Reagents

All tissue culture reagents and media were purchased from Invitrogen. Recombinant proteins (CD4, gp120) and human monoclonal antibodies (anti-gp120, anti-CD4) and AZT were obtained through the AIDS Research and Reference Reagent Program (ARRRP), National Institute of Allergy and Infectious Diseases (NIAID) (Bethesda, MD, USA). Peptides were custom synthesized from United Peptides Ltd., USA. All the stock reagents were dissolved in endotoxin-free water. Unless otherwise stated, all other chemicals and media were of high quality and procured from local suppliers.

### Cell Lines

TZM-bl cells (Hela cells expressing CD4, CCR5 and CXCR4) carrying luciferase and β-galactosidase (β-Gal) genes, downstream to HIV-1 LTR promoter [5], CEM-GFP cells, HL2/3 cells (expressing HIV-1 envelope protein, gp120) and H9 cell lines were procured from ARRRP, NIAID, USA. HEK-293T cells were received as a gift from Dr. Debashish Maitra, National Centre for Cell Science (NCCS), Pune, India. HeLa cells were procured from NCCS, Pune, India. Immortalized human vaginal epithelial cells (VK2/E6E7) were kindly gifted by Dr. Raina Fichorova, Brigham Women’s Hospital, Harvard Medical School, Boston, USA.

HL2/3, HEK293T, HeLa and TZM-bl cells were propagated in Dulbecco’s Modified Eagle’s Medium (DMEM) with 10% fetal bovine serum (FBS) (Invitrogen, USA), 1% penicillin/streptomycin. CEM-GFP and H9 cells were maintained in RPMI containing 10% FBS, 1% penicillin/ciprofloxacin (Sigma-Aldrich, St. Louis, USA). VK2/E6E7 cells were maintained in keratinocyte serum-free medium (KSFM) supplemented with 50 μg/ml of bovine pituitary extract (BPE), 0.1 ng/ml epidermal growth factor (EGF), 100U/ml penicillin, and 100 μg/ml of streptomycin. PBMCs were isolated from heparinized whole blood by Ficoll density gradient centrifugation and cultured in RPMI containing 10% FBS and 20U/ml Interleukin-2. PBMCs were activated with 5μg/ml phytohemagglutinin (PHA), 48 hours prior to HIV infection. All cell lines were maintained at 37°C in a humidified atmosphere containing 5% CO_2._

### Plasmids, Viruses and Viral stocks

HIV-1-IIIB and HIV-1 BaL strains were obtained from ARRRP, NIAID, USA. *p*NL4-3 and *p*ADA plasmids were provided by Dr. Debashish Maitra, NCCS, Pune, India. VSV-G construct (pHCMV-G) and Env defective construct (R9∆E) were kind gifts from Prof. Christopher Aiken, Vanderbilt University Medical Centre, Nashville, USA.

Viral stocks of HIV-1 IIIB and HIV-1 BaL were prepared in immortalized H9 cells. H9 cells (5x10^5^) were infected with viral strains and supernatants were collected on day 4, 7, 11, 15 and 22 for p24-based antigen assay (XpressBio) to quantitate viral stocks. HEK-293T cells were used to generate viral stocks following transfection with *p*NL4-3 and *p*ADA plasmids. HEK-293T cells (3x10^5^) were transfected with 5μg of plasmid DNA (*p*NL4-3 and *p*ADA) using Lipofectamine-2000. HIV-1 (VSV-G) pseudotyped virus, referred to as HIV-1 VSV, was produced by co-transfection of HEK-293T cells with 5ug each of R9∆E and pHCMV-G plasmids. Cell-free viral supernatants were collected after 48 hrs and stored at -80°C in 100 μl aliquots. p24 levels of pseudotyped virus in the supernatant was determined by p24 antigen ELISA kit. Infectivity of viruses was checked by measuring β-gal activity in TZM-bl cells. Multiplicity of Infection (MoI) was calculated and 0.1 MoI was used for various anti-HIV assays.

### **Determination of Multiplicity of Infection** (**MoI)**

TZM-bl cells (1x10^5^) were seeded 24 hrs prior to the infection with viral stocks. Cells were infected with three dilutions (0.1μl, 0.5μl and 1μl) of HIV-1 NL4-3, HIV-1 IIIB, HIV-1 BaL and HIV-1 ADA5 for 6 hrs. Cells were washed with fresh medium and incubated for another 48 hrs; cells were washed and fixed with 0.25% glutaraldehyde in PBS for 10 min. This was followed by washing with PBS and X-gal staining. Blue foci were counted in 5 different fields within 12 hrs of staining. Infective virions/ml was calculated using the formula: Total number of blue foci / virions = No. of blue foci x surface area conversion factor (75 for a 24 well plate) x dilution factor.

### *In-silico* design of Hb-α derived peptides

Protein sequence of HIV-1 gp120 (accession number P04578) was retrieved from Swissprot database [20]. The gp120 structure (1GC1) [21] in non-glycosylated state was acquired from the Protein Data Bank (PDB) [22]. Since, no reported crystal structure of glycosylated gp120 of HIV-1 was available, we looked for glycosylated gp120 proteins of other retroviruses. Thus, a thorough search for the same listed Simian Immunodeficiency Virus (SIV) with PDB id: 2BF1 [23] and a resolution of 4.0Å. This is reported to be an unliganded gp120. This protein sequence was aligned against HIV-1 gp120 using T-Coffee software [24] to identify the percentage of residual identity. Further, based on the identity score, this was considered as the template for modelling of HIV-1 gp120 using Modeller 9.10 software [25]. Twenty structures were generated. Of these, structure with the least DOPE (Discrete optimized Protein Energy) score was selected. Their steric clashes were removed using SwissPdb Viewer [26]. Similar protocol was followed for generating the structures of human Hb peptides and their analogues using 2RAO as the template [27]. Recently the 3D structure of glycosylated HIV gp120 has been deposited in Protein Data Bank with PDB id: 4RQS and a resolution of 4.49Å in liganded state [28]. Here, we considered this HIV gp120-CD4 complex (as liganded state) for docking with peptides.

### Docking studies

Both gp120 (unliganded and liganded) and peptides were docked using Cluspro 2.0 server [29]. This online server uses supercomputing facilities to preprocess the receptor and the peptides wherein both the structures undergo predocking minimization before docking. The generated structures for gp120 and Hb derived peptides were clustered and ranked according to their cluster size. The structure with the maximum cluster size was chosen for this study. Finally the selected docked poses were considered for the protein-protein interaction analysis through DCOMPLEX software [30]. Binding interactions between gp120 and peptides were visualized using CHIMERA visualization software [31].

### Synthesis of peptides

Based on prediction algorithm, Hb-α subunit derived peptides that showed binding affinities to CD4 binding site of gp120 were commercially synthesized as C-terminal amides by Fmoc solid-phase synthesis. A 25 mer scrambled peptide (sHbAHP-25 or sP) was also synthesized. Purity of all the peptides was more than 90%. The homogeneity analysis was carried out by standard mass-spectrometry (MS) method.

### Development of anti-peptide antibodies

To raise anti-peptide polyclonal antibody, HbAHP-25 was coupled with a carrier protein, keyhole limpet hemocyanin (KLH) (Pierce, Rockford, IL, USA) according to manufacturer’s instructions. Two rats were subcutaneously immunized with 100 μl of PBS containing 50μg of the peptide emulsified in 50 μl of complete Freund’s adjuvant. The first immunization was followed by two boosts at 2-week intervals with 50μg of the peptide emulsified in incomplete Freund’s adjuvant. Anti-serum was collected 10 days after the last injection and titers were checked by ELISA.

### Cytotoxicity assay and selectivity index

The viability of PBMCs, TZM-bl, VK2/E6E7, and CEM-GFP cells was determined at 24 or 120 hr, after the addition of HbAHP-25, by MTT assay [32]. Briefly, PBMCs, VK2/E6E7, TZM-bl, and CEM-GFP cells (5x10^4^ for 24 hr assay and 2x10^4^ cells for 120 hr assay) were seeded in a 96-well microtiter plate 24 hrs prior to the treatment with peptides. Cells were treated with different concentrations of peptides (4.4–1142.4μM). MTT (10 μl of 1mg/ml) was added to each well, followed by addition of 100 μl of DMSO (Sigma-Aldrich) after 3 hrs. The absorbance was measured at 540nm using ELISA reader (ELX-800, BioTek Instruments Ltd, USA.). Untreated cells were used as medium control and 0.1% Triton X-100 treated cells served as a positive control. The 50% cytotoxicity (CC_**50**_) value was defined as the concentration of HbAHP-25 that reduced the absorbance of treated cells by 50% as compared with control cells.

### Enzyme Linked Immunosorbent Assay (ELISA)

ELISA was carried out to determine whether HbAHP-25 binds with gp120. Briefly, wells of a 96-well microtiter plate were coated with 100 μl of two fold serially diluted HbAHP-25 (2.2–71.40 μM) or sP, for 16 hrs at 4°C. After blocking with 3% bovine serum albumin (BSA) in PBS for 30 min, 100 μl of gp120 (100ng/well) in PBS-0.05% BSA was added to each well and incubated for 2 hrs at 37°C. After three washings with PBS-Tween-20 (PBS-T), cells were incubated with anti-gp120 antibody at 1:200 dilution and then with horse radish peroxidase-(HRP) conjugated secondary goat anti-human antibody (1:10000 dilution) for 1 hr at 37°C. This was followed by incubation with O-phenylenediamine for 20 min. The absorbance was measured at 492 nm on a microplate ELISA reader (ELx-800, Bio-Tek Instruments, USA). Background values obtained in the wells without HbAHP-25 were subtracted from the absorbance values from wells having all other reagents.

### Competitive ELISA

96 well microtiter plate was coated with soluble gp120 (1μg/well) from HIV-1 BaL and HIV-1 IIIB strains for 16 hrs at 4°C. Following blocking with 3% BSA, wells were treated with HbAHP-25 for 30 min and then with recombinant CD4 protein for 1 hr. Anti-CD4 monoclonal antibody (1:200) was added to the wells, followed by HRP-labeled goat anti-human secondary antibody (1:10000). Rest of the protocol followed was same as described above.

To determine the binding site of HbAHP-25 on gp120, competitive ELISA was carried out using mAbs VRC01 and F105 which bind to the CD4 binding site of gp120; and mAbs 2G12 and 17b which bind outside the CD4 binding domain of gp120. MaxiSorp flat bottom 96 well plate was coated with gp120 (1μg/well) as mentioned above. Wells were treated with monoclonal antibodies (VRCO1/F105/2G12/17b) to gp120 (5μg/ml) for 1 hr. After washing, HbAHP-25 (35.7μM) was added. Binding of HbAHP-25 to gp120 in the presence or absence of gp120 antibodies was detected by adding anti-HbAHP-25 antibody (1:400) for 1 hr at RT. After washing, HRP-labeled goat-anti rat secondary antibody (1:8000) was added. Background absorbance was subtracted from the wells that were treated with all the reagents except HbAHP-25. Similarly, gp120 coated wells were treated with various concentrations of VRC01 and F105, and then treated with HbAHP-25. Rest of the protocol was same as above.

### Surface Plasmon Resonance

The binding of HbAHP-25 or scrambled peptides to gp120 was also analyzed by surface plasmon resonance (SPR) using a BIAcore 3000 (BIAcore Inc., Piscataway, NJ) as described earlier [33]. Briefly, gp120 (20 μg/ml) was immobilized onto carboxymethylated dextran (CM5) surface based sensor chip by amine coupling method according to the manufacturer’s instructions. Different concentrations of HbAHP-25 made in HBS-EP buffer (pH7.4) were passed over gp120 coated sensor chip at a flow rate of 30 μl/min with an association phase of 5 min and dissociation phase of 5 min during which buffer alone was perfused. 10mM NaOH and 0.5% SDS were used to regenerate the sensor chip surface after each experimental cycle. The binding response in resonance units (RU) was recorded as a function of time. Non-specific binding was subtracted from the control flow cells without gp120. Binding kinetics was determined with BIA evaluation 4.1 software (BIAcore) using 1:1 Langmuir binding model.

### Anti-HIV activity of HbAHP-25 by p24 antigen assay

CEM-GFP cells and PHA stimulated PBMCs (2x10^5^ cells/well) were infected with four strains of HIV-1 (IIIB, BaL, ADA5 and NL4-3). Viral particles (MoI-0.1 U) were pre-incubated with HbAHP-25 (4.46–71.40 μM) or sP (71.40 μM) for 1 hr and then added to cells. Cells were washed after 6 hrs and cultured for 120 hrs in fresh media containing HbAHP-25 or sP. Supernatants were collected and accumulated p24 levels were determined by sandwich ELISA as per the manufacturer’s protocol (XpressBio, Thurmont, MD, USA). AZT (10nM) was used as positive control for inhibition. For GFP analysis, CEM-GFP cells were observed under fluorescent microscope (Carl Zeiss, Oberkochen, Germany) and photographed using a digital camera (DSC–S75; Sony, Tokyo, Japan). IC_**50**_ was defined as the concentration that inhibited 50% of viral activity. The selectivity index (SI) was evaluated as the ratio of CC_**50**_ to IC_**50**_ (SI = CC_**50**_/ IC_**50**_).

### Anti-HIV activity of HbAHP-25 by Luciferase assay

TZM-bl cells (2x10^4^cells/well) were infected with different HIV-1 strains. Viruses at a concentration of 0.1 MoI were pre-incubated with HbAHP-25 (4.46–71.40 μM) or sP (71.40 μM) for 1 hr at 37°C, and allowed to infect TZM-bl cells for 6 hrs at 37°C. After washing, 100μl of fresh DMEM supplemented with 10% FBS and 1% Penstrep was added and cells were cultured for another 48 hrs. Luciferase activity of TZM-bl cells was measured using Luminometer, after the addition of luciferase substrate (Bright-Glo) as reported earlier [34].

### Time-of-Addition Assay

TZM-bl cells (3x10^4^) grown in a 96 well plate for 24 hrs, were infected with HIV-1 IIIB and ADA5 (at 0.1 MoI). HbAHP-25 or sP (71.40 μM) was added at different stages of infection viz: a) HIV-1 strains were pre-incubated with the peptides for 1 hr at 37°C and added to cells b) TZM-bl cells were treated with mixture of HIV-1 virus and peptide c) TZM-bl cells were first infected with HIV-1 virus and after 6 hrs incubated with the peptides and d) Peptides were present at all the three steps i.e pre-incubation, during infection and post infection. Luciferase activity was measured at 48 hrs post treatment using Luminometer (ELx-800, Bio-Tek Instruments, USA). VRC01 (0.1 μM) antibody was used as a positive control.

### Flow Cytometry Analysis

In order to assess whether the binding of HbAHP-25 to gp120 is specific, flow cytometric analysis was carried out using three different cell lines. HL2/3 cells (3x10^5^), TZM-bl cells (3x10^5^) and HeLa cells (3x10^5^) (negative controls) were treated with 35.7μM HbAHP-25 for 1 hr at RT, and then washed with FACS buffer (0.2% FBS in PBS). Cells were incubated with anti-HbAHP-25 antibody (1:400) for 30 min at RT, and washed again with the FACS buffer. After washings, cells were incubated with FITC labeled goat anti rat antibody (1:2000) for 20 min at RT. Cells were washed with same buffer twice and binding was evaluated using flow cytometer (BD Accuri C6, USA). The data was acquired and analyzed on BD Accuri software.

### Effect of HbAHP-25 on HIV-1 pseudotyped Virus (HIV-1 VSV):

HIV-1 NL4-3 viral stocks were generated by transfecting 293T cells with HIV-1 NL4-3 (wt) and co-transfecting pHCMV-G and R9∆E in 293T cells. Viral supernatants were collected and p24 levels were measured. To determine viral infectivity, CEM-GFP cells (1x10^5^) were infected with both viruses (50ng/ml p24) for 4 hrs, washed and cultured for 3 days. Supernatants were collected and p24 levels were measured.

Effect of HbAHP-25 on entry and replication of HIV-1 (VSV) virus in CEM-GFP cells was investigated. CEM-GFP cells were activated with 5μg/ml PHA for 48 hrs before the infection. CEM-GFP cells (1x10^5^) were infected with HIV-1 NL4-3 (wt) or HIV-1 (VSV) (20ng/ml) for 4 hrs in the presence and absence of HbAHP-25 (35.7μM). Cells were washed twice to remove the unbound virus. Supernatants were collected at day 4 post infection and p24 levels were determined. VRC01 (0.1μM) was used as a control. CEM-GFP cells were simultaneously infected with HIV-1 (wt) and HIV-1 (VSV) (20ng/ml p24) for 1 hr and entry of these viruses into cells was measured in the presence and absence of HbAHP-25. Cells were washed three times with PBS, lysed and intracellular p24 was assessed by p24 ELISA (Xpress Bio).

### Anti-HIV activity of HbAHP-25 in the presence of seminal plasma and vaginal fluid

The anti-HIV efficacy of HbAHP-25 was tested in the presence of seminal plasma and vaginal fluid as described earlier [35, 36]. Briefly, semen was obtained from four healthy, HIV-seronegative individuals. Seminal plasma was collected from semen samples and incubated with different concentrations of HbAHP-25 for 10 min at RT. HIV-1 IIIB and HIV-1 ADA5 strains (0.1 MoI) were then added to the mixture and incubated for 1 hr. TZM-bl cells were incubated with the mixture of seminal plasma, peptide and virus for 4 hrs. Removal of media containing seminal plasma after the incubation was critical to avoid cytotoxicity. Cells were washed with fresh DMEM and allowed to grow for 48 hrs. Luciferase activity was measured using Luminometer.

The anti-HIV activity of HbAHP-25 was also determined in the presence of vaginal fluid [37]. Briefly, vaginal fluid samples were collected from three healthy, HIV-negative and filtered with 0.22μm filter. Samples were mixed with HbAHP-25 for 10 min. Rest of the protocol followed was same as described above for seminal plasma samples. Alternatively, 35.7μM of HbAHP-25 was incubated with seminal plasma or vaginal fluid samples for different time periods. 0.1 MoI of HIV-1 IIIB and HIV-1 BaL were then incubated with this mixture for 1 hr and then added to TZM-bl cells. Cells were washed after 4 hrs and cultured for 48 hrs. Anti-HIV activity of HbAHP-25 was then evaluated as above.

### Circular Dichorism (CD) Spectroscopy

CD spectra was performed on a JASCO CD Spectrometer (J-815) at 37^°^C using a 5.0-nm bandwidth, 0.1-cm path length, 5-s response time, and a 10-nm/min scanning speed. 10 μM of HbAHP-25 dissolved in PBS (10mM; pH 7) and sodium acetate buffer (10mM; pH 4) were acquired and base line corrections were done by subtraction of a blank corresponding to the solvent. The α-helical content of HbAHP-25 was calculated from the CD signal by dividing the mean residue ellipticity at 222 nm by the value expected for 100% helix formation.

### Statistical Analysis

All the statistical analysis was done using GraphPad Prism software version 5.0 (GraphPad software, CA, USA). All values were expressed as the mean ± standard deviation (SD) of six observations from three independent experiments. The values were evaluated using the two-tailed unpaired Student’s t-test, the following notations have been used to denote statistical significance in the figures: *: P< 0.05; **: P < 0.01 and ***: P < 0.001 and considered to be significant if the ‘p’ value was less than 0.05 (*p<0*.*05*).

## Results

### Hb-α peptides interact with CD4 binding domain of gp120

Structure of HIV-1 gp120 was modelled as described in materials and methods. The query sequence, HIV-1 gp120, was aligned against SIV gp120 using T-Coffee software. The result showed 38.30% residual identity. Based on this identity report, the HIV-1 gp120 was modelled using Modeller software. Three Hb-α subunit derived peptides, with a potential to bind gp120 of HIV-1, were also designed using similar *in silico* approach. All these modelled structures were selected based on their least DOPE scores and considered for docking studies.

For unliganded protein-peptide interactions, when we compared the gp120-peptide interactions with gp120-CD4/X5-antibody [38] interaction, it was observed that the peptide-1, its analogues and peptide-2 interact at regions proximal to CD4 binding site of gp120. Peptide-3 preferred to dock to the pocket and the flanking residues. Peptide-1 showed higher binding energies than Peptide 2 and Peptide 3. Next, when we compared unliganded gp120-peptide interactions with liganded gp120-peptide interactions, we observed that peptides showed better binding with unliganded gp120 than the liganded gp120 which is evident by their binding energies, as shown in S1 Table.

### Confirmation of *in-silico* data by binding ELISA and p24 antigen assay

Of the three peptides, peptide-1 and peptide-3 showed binding to gp120 of HIV-1 IIIB as shown in S1 Fig. However, peptide-1 showed greater binding as well as anti-HIV activity than peptide-3. As indicated in S2 Fig, peptide-2 did not show any anti-HIV-1 activity.

**Fig 1:**
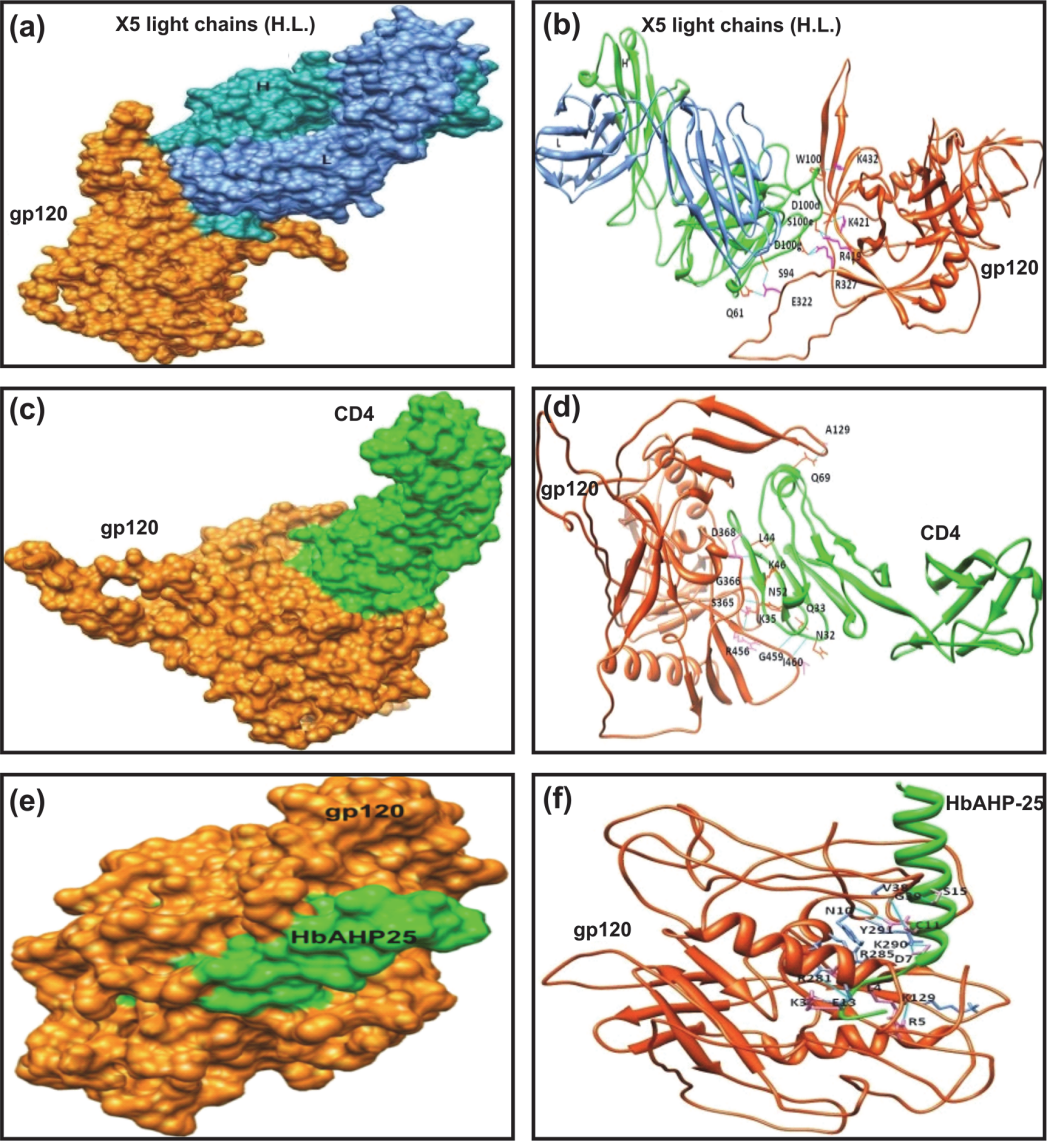
Docking of HbAHP-25 with glycosylated gp120 of HIV-1. Structure of HIV-1 gp120 and HbAHP-25 were modelled with modellar software and docked using Cluspro 2.0 server. Docked pose of gp120 with X5 in surface generated (a) and ribbon format (b); both H and L chains are shown in the figure with their residual interactions as reported in 1GC1. Docked pose of gp120 with CD4 in surface generated (c) and ribbons format (d). Docked pose of gp120 with HbAHP-25 in surface generated (e) and ribbons format (f). The helical peptide is shown in green (bold).

In order to improve gp120 binding ability, two analogues of peptide-1 were generated. In the first one, amino acid at position 24 i.e. alanine was replaced by arginine (A24R) and in the other analogue, phenylalanine at position 11 by cysteine (F11C) and alanine at position 24 by arginine (A24R), so as to increase net positive charge and possibly binding to gp120. The analogue of peptide-3 was also made, where four amino acids were replaced with positively charged arginine residues (Table 1). Of the two peptide-1 analogues, analogue-1b showed better anti-HIV activity than analogue-1a and analogue of peptide-3 against HIV-1 IIIB strain, as depicted in S3 Fig Therefore, rest of the studies were carried out to investigate the anti-HIV potential of analogue-1b or HbAHP-25 (Fig 1).

**Table 1:** Hemoglobin-α subunit derived peptides interaction with gp120.

Peptides	Amino acid residues	Peptides sequence	Amino acid change	Anti-HIV activity	Charge	Hydrophobicity
Peptide- 1	25	AHKLRVDPVNFKLLSHCLLVTLAAH (88–112)	_	+	+2	56%
Analogue of peptide-1 (1a)	25	AHKLRVDPVNFKLLSHCLLVTLA**R**H (88–112)	(A24R)	+	+3	52%
Analogue of peptide-1 (1b)	25	AHKLRVDPVN**C**KLLSHCLLVTLA**R**H (88–112)	(F11C) and (A24R)	++	+3	52%
Peptide- 2	29	SAQVKGHGKKVADALTNAVAHVDDMPNAL (52–80)	_	NA	0	44%
Peptide- 3	29	VKGHGKKVADALTNAVAHVDDMPNALSAL (55–83)	_	+	0	48%
Analogue of peptide-3	29	VKGH**R**KKVADAL**RR**AVAHVDDMP**R**ALSAL (55–83)	(G5R, N13R, A14R & N24R)	NA	+4	48%
Scrambled peptide(sP)	25	PLVKDVLRSLCNHAHKCVTLALLRH (for peptide-1) (88–112)	_	NA	+3	52%

Sequences of the designed peptides along with analogues are enlisted in the table. Amino acids indicated in bold-underlined are the changes made in the sequence of peptide-1 and peptide-3 to design their analogues. The regions of human Hb-α from which sequence of amino acids of these peptides was taken, is given in the parenthesis. (+: moderate, ++: significant, NA: No activity)

### HbAHP-25 doesn’t affect cell viability

**Fig 2:**
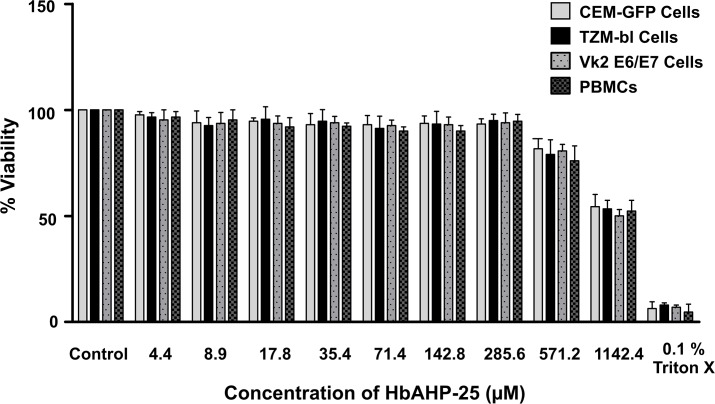
Effect of HbAHP-25 on the viability of TZM-bl cells, VK2 cells, CEM-GFP cells and PBMCs. Cells were treated with HbAHP-25 (4.4–1142.4µM) for 120 hrs. Percentage of viability was calculated with respect to control (not treated with HbAHP-25). 0.1% Triton X was used as a positive control. Values are the mean (± SD) of three replicates.

MTT assay results revealed that HbAHP-25 is not cytotoxic to PBMCs, VK2/E6E7, TZM-bl, and CEM-GFP cells (Fig 2). Viability was approximately more than 90% even when cells were treated with 285.60 μM HbAHP-25. 50% loss in viability of cells (CC_**50**_) was observed at 1142.40 μM concentration of HbAHP-25, which is 5 fold higher than its IC_**50**_. We observed 50% inhibition (IC_**50**_) on all the four viral strains at 35.70 μM of HbAHP-25. The selectivity index (CC_**50**_ / IC_**50**_) of the peptide was 32.

### HbAHP-25 interacts with gp120 of HIV-1

**Fig 3:**
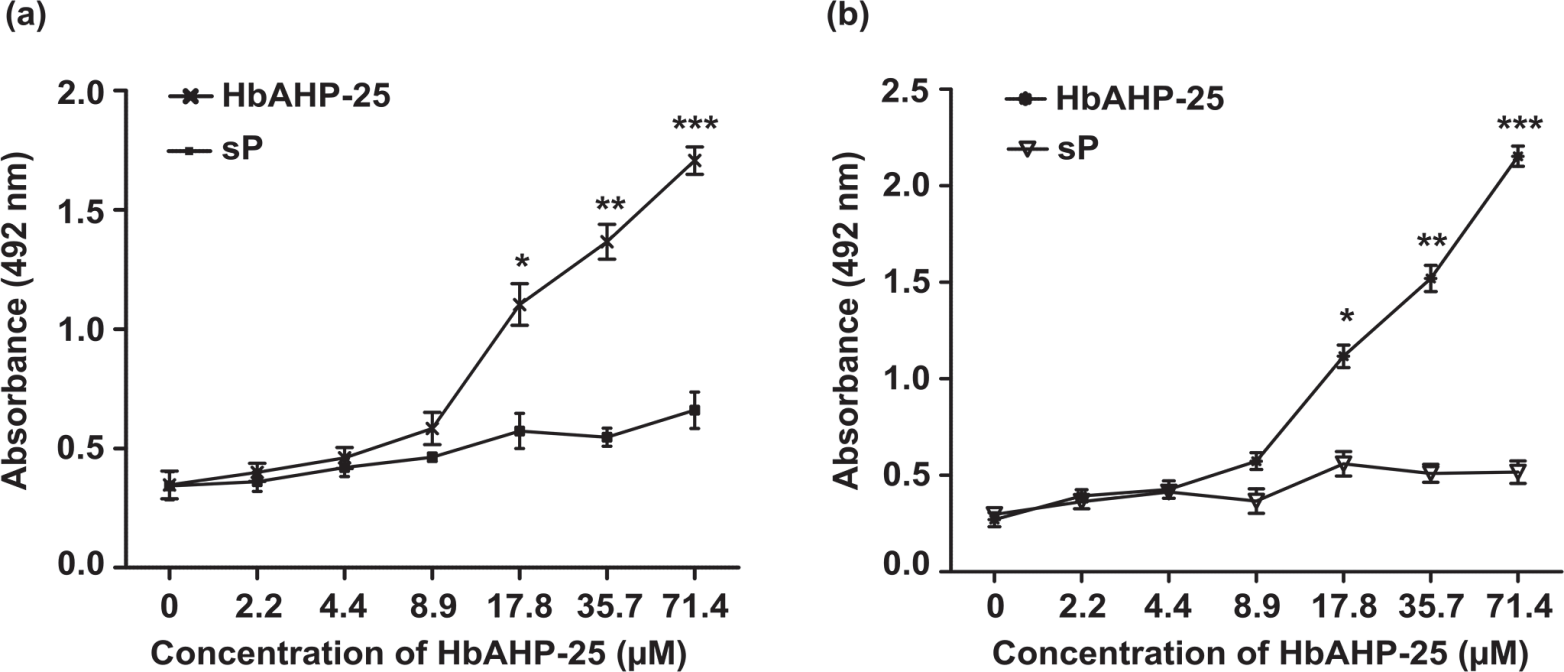
Binding of HbAHP-25 to recombinant gp120 protein. Binding of HbAHP-25 or scrambled peptide (sP) to gp120 of HIV-1 IIIB (a) and HIV-1 BaL (b). Wells coated with various concentrations of HbAHP-25 or scrambled peptide (sP) were incubated with gp120 for 2 hrs at 37°C. This was followed by addition of gp120 antibody and absorbance was read at 492 nm. Background absorbance was subtracted from the wells where only gp120 was added (no HbAHP-25). Each bar represents the mean (± SD) of six observations from three experiments performed on different days (*p<0.05; **p<0.01; ***p<0.001).

HbAHP-25 binds to recombinant gp120 of both HIV-1 IIIB and HIV-1 BaL in a dose dependent manner. We observed significant binding at concentrations above 8.9 μM. As expected, the scrambled peptide did not bind to gp120 of either HIV-1 IIIB (Fig 3A) or HIV-1 BaL (Fig 3B).

### HbAHP-25 has high affinity for binding to gp120

**Fig 4:**
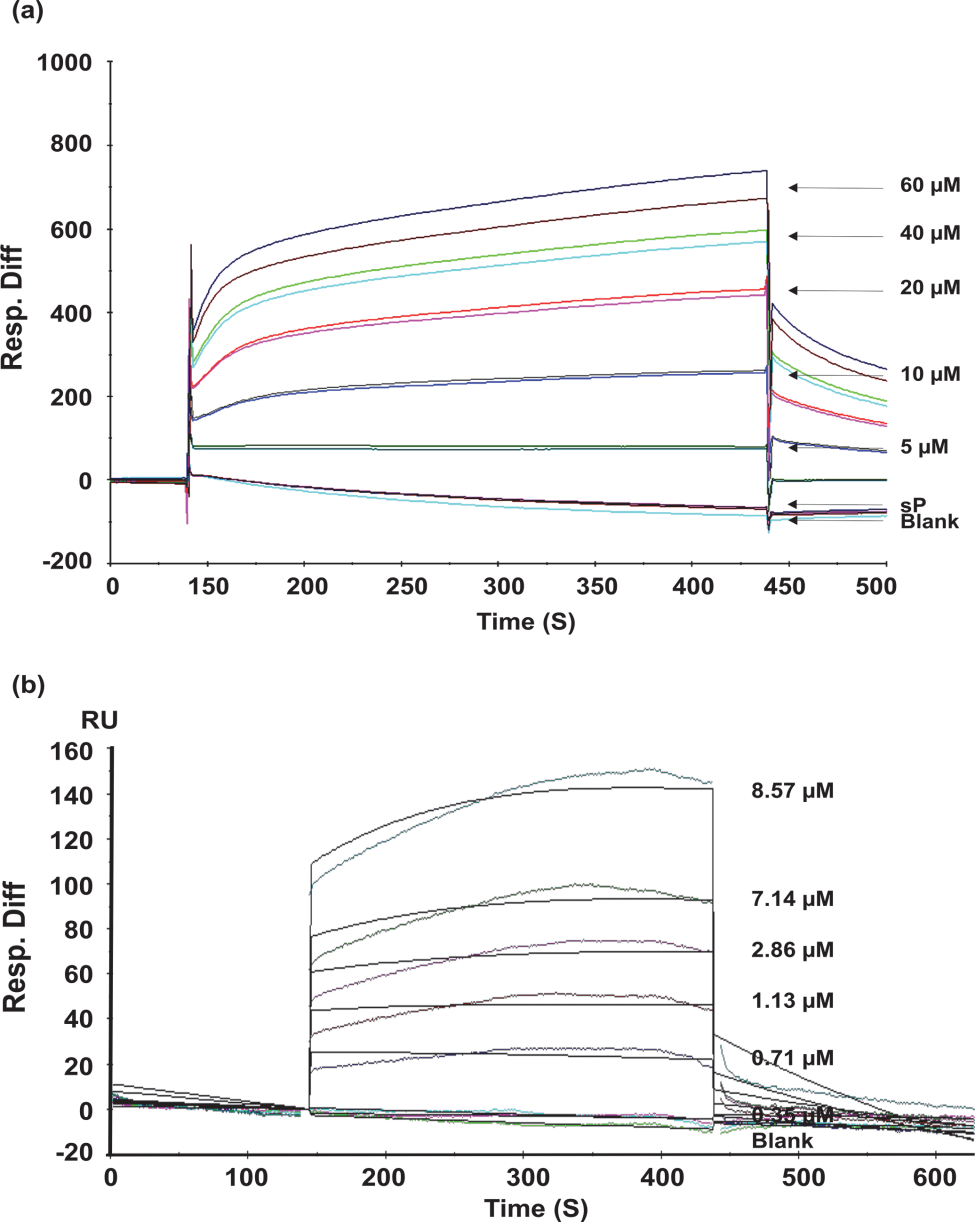
Sensogram showing binding of HbAHP-25 to gp120. a) Different concentrations of HbAHP-25 and sP (20μM) were passed on gp120 immobilized chip. Direct binding was detected, represented as response units (RU). The image is representative of one of three identical experiments performed on three different days. b) Indicated concentrations of HbAHP-25 were passed over CM5 chip immobilized with gp120. Langmuir binding model (1:1) was used to evaluate binding constants. Sensograms are shown in different colors, corresponding to different concentration of HbAHP-25, and fits in black. Injections were carried in duplicates and results were identical.

To determine kinetics of HbAHP-25-gp120 interaction, we performed SPR analysis. Increasing concentrations of HbAHP-25 were passed over the surface immobilized gp120. As shown in Fig 4, we observed direct binding of HbAHP-25 to gp120; the binding was gradual, saturable, and increased with increasing concentrations of HbAHP-25. HbAHP-25 bound to gp120 with high affinity, with an Rmax of 99.7 RU and KD of 6.75 μM (Table 2). These results clearly indicate that HbAHP-25 has significant binding affinity with gp120.

**Table 2:** Rate and equilibrium constants for binding of HbAHP-25 to gp120.

Ka (M^-1^ S^-1^)	Kd (S^-1^)	KD (M)	Chi2
1.25x10^3^	8.47x10^-3^	6.75x10^-6^	14.7

Ka and Kd are rate constants for association and dissociation respectively; KD is the equilibrium dissociation constant

### HbAHP-25 binding is specific to cells expressing gp120

**Fig 5:**
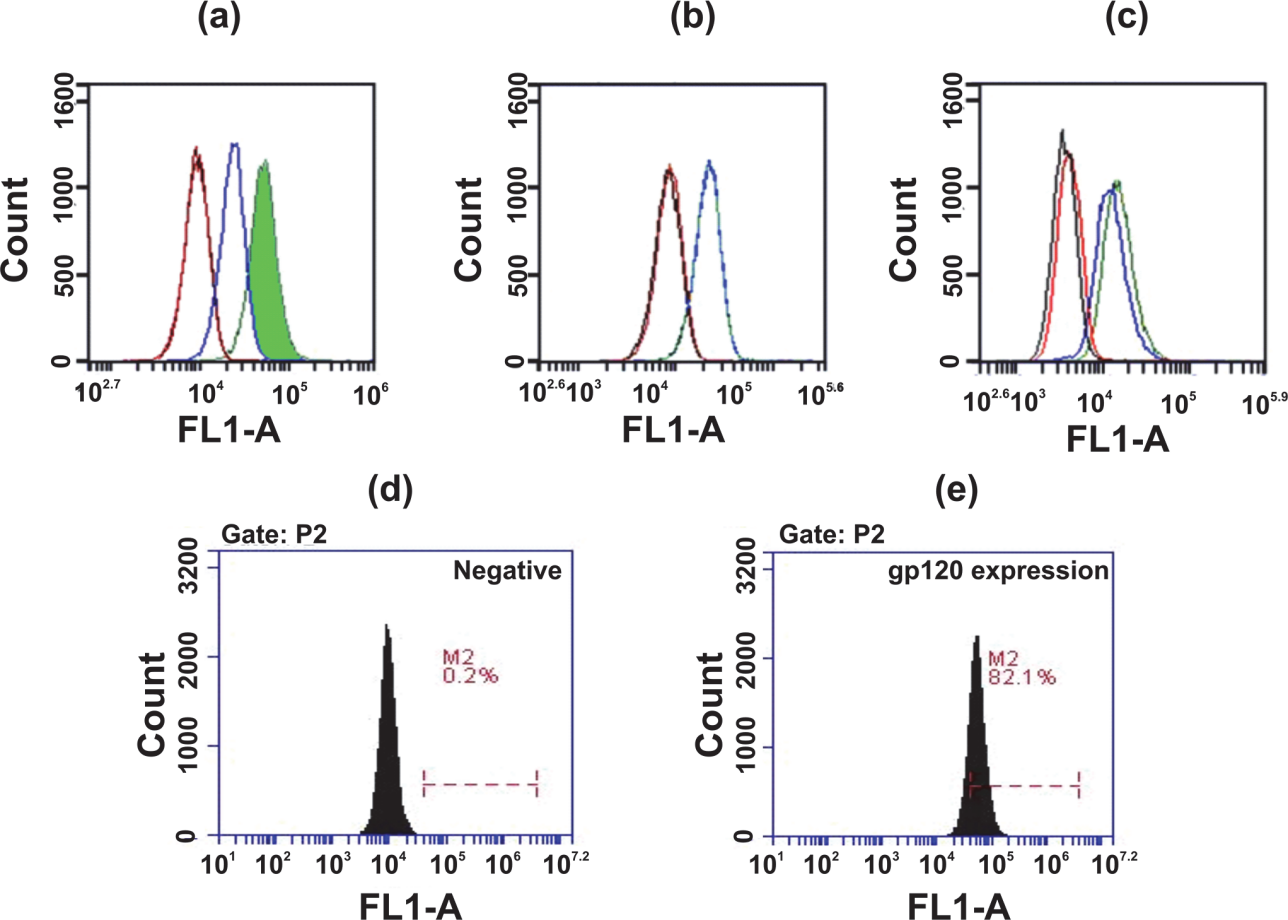
Binding of HbAHP-25 to cellular gp120. Offset histograms of cells (a) HL2/3 cells (b) TZM-bl cells and (c) HeLa cells indicate the extent of fluorescence intensity observed in FL-1A channel. Cells were treated with HbAHP-25 for 1 hr at RT, and then incubated with anti-HbAHP-25 antibody for 30 min. This was followed by addition of FITC labeled goat anti-rat antibody. Red lines correspond to the fluorescence of untreated group i.e only cells. The black lines correspond to the primary antibody control, while as blue lines correspond to the secondary antibody (FITC labeled) control, where both primary and secondary antibody was added to cells. The green line is the test group, and signifies the fluorescence in the peptide-treated group. The figure is representative image of one of the three independent experiments. (d, e) Expression of gp120 on HL2/3 cells (e). HL2/3 cells were treated with 2G12 mAb (500ng/ml), followed by anti-human (FITC labeled) secondary antibody. Cells not treated with 2G12 were used as a negative control (d).

To determine whether HbAHP-25 binds specifically to gp120 present on the cell surface, flow cytometric analysis was carried out using HL2/3 cells expressing gp120. HbAHP-25 specifically binds to HL2/3 cells expressing gp120, which was evident by shift in fluorescent peak in FL-1A channel (60.10 ± 4.3%). This shift in peak was not evident for TZM-bl and Hela cells (<3%), which do not express gp120 (Fig 5). These results demonstrated that HbAHP-25 binds to gp120 and not to the other cell surface proteins.

### HbAHP-25 inhibited CD4 binding to gp120

**Fig 6:**
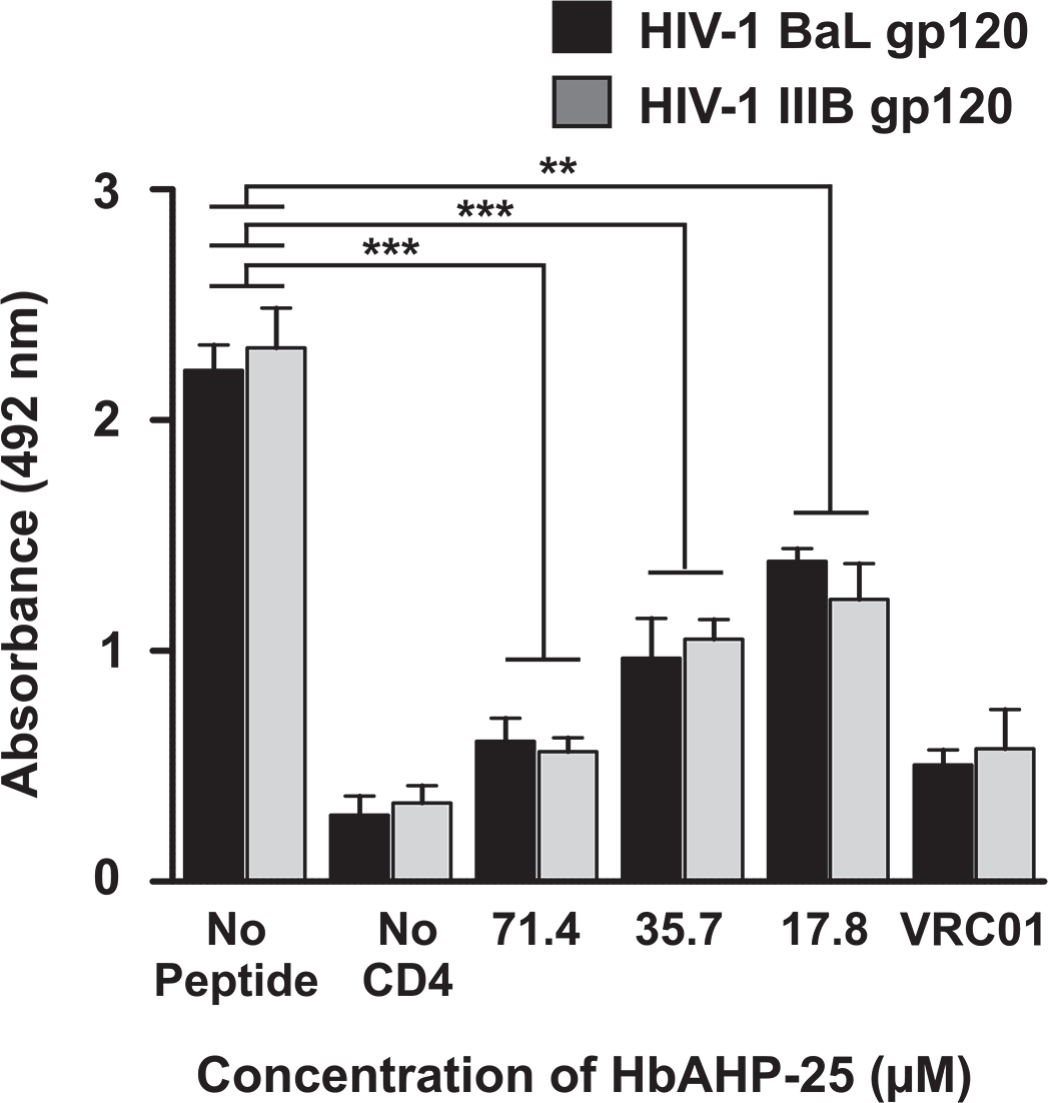
Inhibition of CD4 binding to gp120 by HbAHP-25. gp120 coated wells were incubated with HbAHP-25 for 30 min and then with recombinant CD4 for 1 hr. Following this, wells were incubated with anti-CD4 monoclonal antibody for 1 hr and then HRP-labeled goat-anti human secondary antibody was added. Wells not treated with HbAHP-25 were used as a control (No Peptide), and 0.1μM VRC01 antibody was used as a control to inhibit CD4 binding. Wells not having CD4 show the background absorbance (No CD4) (**p<0.01; ***p<0.001).

Competitive ELISA was carried out to analyze whether the interaction between HbAHP-25 and gp120 blocks CD4 from binding to gp120. Results revealed that HbAHP-25 competes with CD4 for binding with gp120 (Fig 6). We observed a significant reduction in the absorbance after CD4 addition to the gp120 coated wells, pre-incubated with HbAHP-25 compared to the wells in which the pre-incubation step with HbAHP-25 was omitted. This indicates that HbAHP-25 binds to either CD4 binding domain of gp120 or to the overlapping region. In brief, the set of experiments established that HbAHP-25 interferes with the binding of gp120 to CD4.

**Fig 7:**
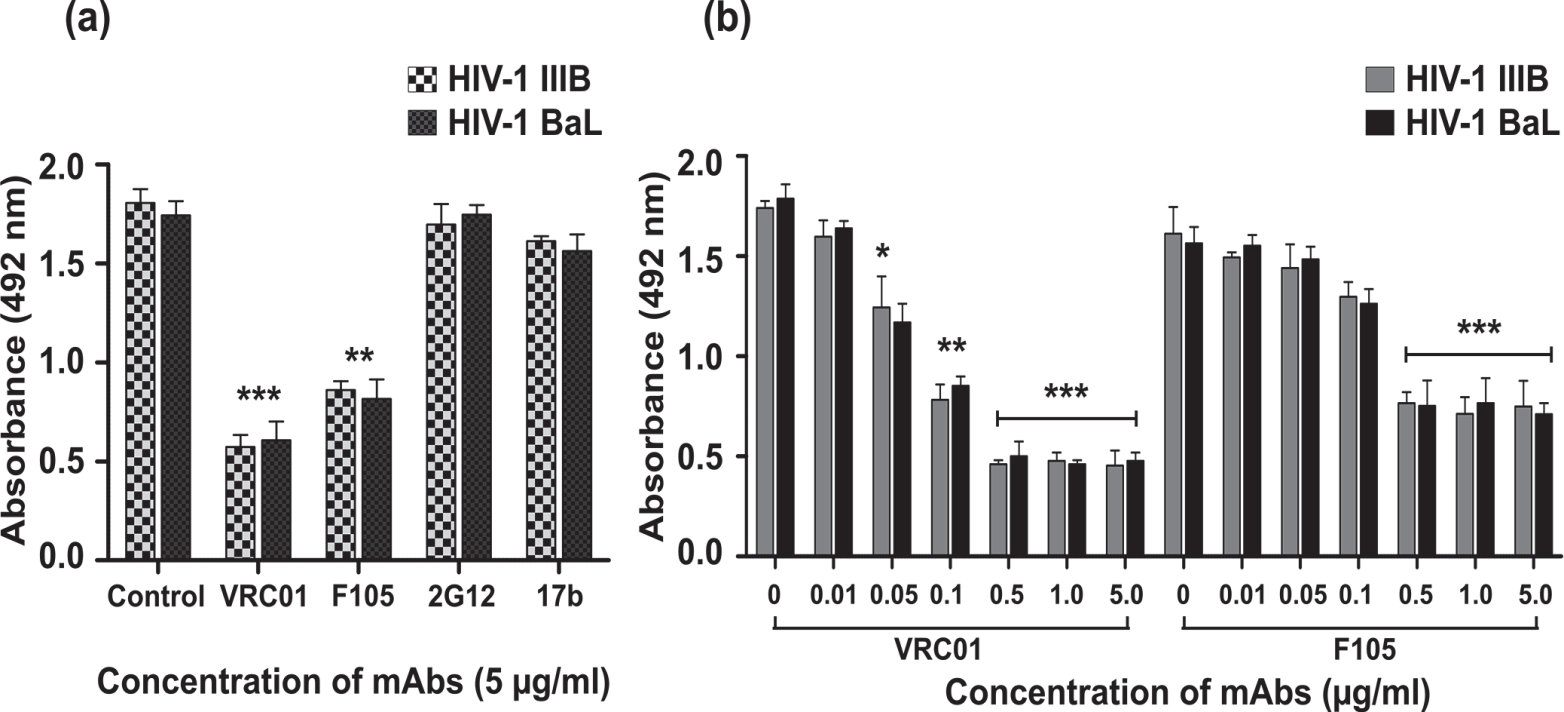
Epitope overlap of different mAbs and HbAHP-25 on gp120. a) Wells coated with gp120 were treated with indicated concentrations of VRC01, F105, 17b and 2G12 for 1 hr. Following this, HbAHP-25 and anti-HbAHP-25 antibody were added sequentially. Wells not treated with any antibody were used as a control. b) gp120 coated wells were incubated with different concentrations of VRC01 and F105, followed by HbAHP-25 and anti-HbAHP-25 antibody as above. Values are the mean (± SD) of three different biological replicates performed on different days (sP: scrambled peptide) (*p<0.05; **p<0.01; ***p<0.001).

Next, we evaluated whether there is any epitope overlap between the binding sites of HbAHP-25 and four anti-gp120 mAbs VRC01, F105, 17b and 2G12. VRC01 and F105 inhibited binding of HbAHP-25 to gp120, whereas 17b and 2G12 (antibodies that do not recognize CD4 binding site of gp120) had minimal effect on binding of HbAHP-25 to gp120 (Fig 7A). This suggested that HbAHP-25, VRC01 and F105 bind to the similar sites on gp120. However, complete inhibition of HbAHP-25 binding to gp120 was not observed even when VRC01 and F105 were used at higher concentrations (Fig 7B). This indicated that gp120 binding sites of VRC01, F105 and HbAHP-25 are somewhat distinct. These findings further hint that HbAHP-25 binds to a region that is proximal to the CD4 binding region on gp120.

### HbAHP-25 is effective against various strains of HIV-1

**Fig 8:**
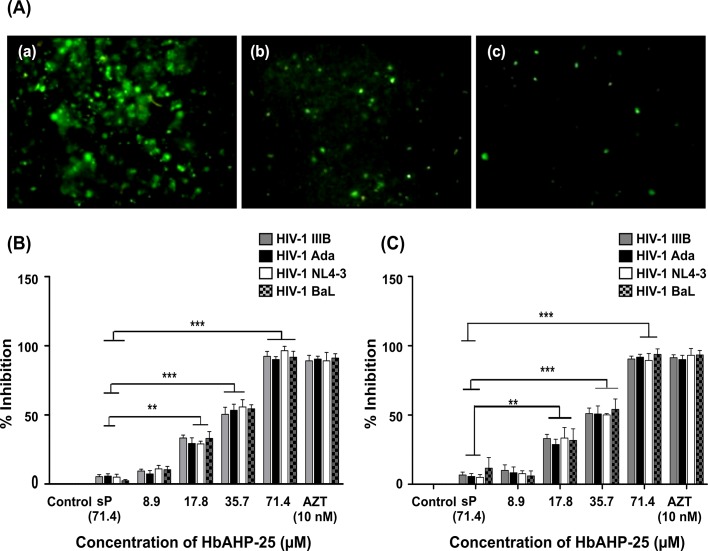
Effect of HbAHP-25 on HIV-1 infection. (A) GFP expression in CEM-GFP cells infected with HIV-1 NL4-3 in the presence or absence of HbAHP-25. Viral strains (0.1 MoI) pre-incubated with different concentrations of HbAHP-25, were used for infecting CEM-GFP cells. GFP expression was monitored on day 5 post infection. (a) GFP expression in the absence of HbAHP-25. (b) GFP expression in the presence of 35.70 μM HbAHP-25 and (c) GFP expression in the presence of AZT (10 nM). (B) p24 antigen levels in HIV-1 infected PBMCs. PBMCs were infected with viral strains pre-incubated with different concentrations of HbAHP-25 or cells infected with viral strains not pre-incubated with HbAHP-25 (100% infection). AZT (10 nM) was used as a positive control. (C) Luciferase activity in TZM-bl cells, infected with viral strains pre-incubated with indicated concentrations of HbAHP-25. Luciferase activity in the cells infected with viral strains not treated with HbAHP-25 was considered as 100% infection. 10 nM AZT was used as a positive control. Background luciferase activity of TZM-bl cells was subtracted from all the wells and % inhibition was calculated accordingly. Values are the mean (± SD) of three different biological replicates performed on different days (**p<0.01; ***p<0.001).

We next evaluated anti-HIV activity of HbAHP-25 using three different assay systems viz; 1) GFP levels in CEM-GFP cells (Fig 8A),2) p24 antigen levels in PBMCs (Fig 8B), and 3) by luciferase reporter assay in TZM-bl cells (Fig 8C).

TZM-bl cells were infected with four HIV-1 strains at 0.1 MOI in the presence of various concentrations of HbAHP-25. Luciferase activity was found to be significantly reduced in the presence of HbAHP-25 (Fig 8C). HbAHP-25 showed significant anti-HIV activity at concentration above 17.80 μM. At 71.40 μM, HbAHP-25 showed about 90% inhibition compared to that seen in untreated cells. This trend was evident in cells infected with all four strains of HIV-1. The IC_50_ value of HbAHP-25 for four different HIV-1 strains was found to be ~35.70 ± 5.23 μM.

Further, PBMCs infected with the HIV-1 strains showed a significant decline in the levels of p24, in the presence of HbAHP-25, compared to the cells infected in the absence of HbAHP-25. More than 90% reduction in p24 levels was observed in cells infected with viral strains in the presence of HbAHP-25 (Fig 8B). GFP expression was also found to be reduced when the CEM-GFP cells were incubated with HbAHP-25 prior to infection (Fig 8A). Thus, all three assays indicated that HbAHP-25 has a significant anti-HIV activity.

### HbAHP-25 inhibits early steps of HIV entry into cells

**Fig 9:**
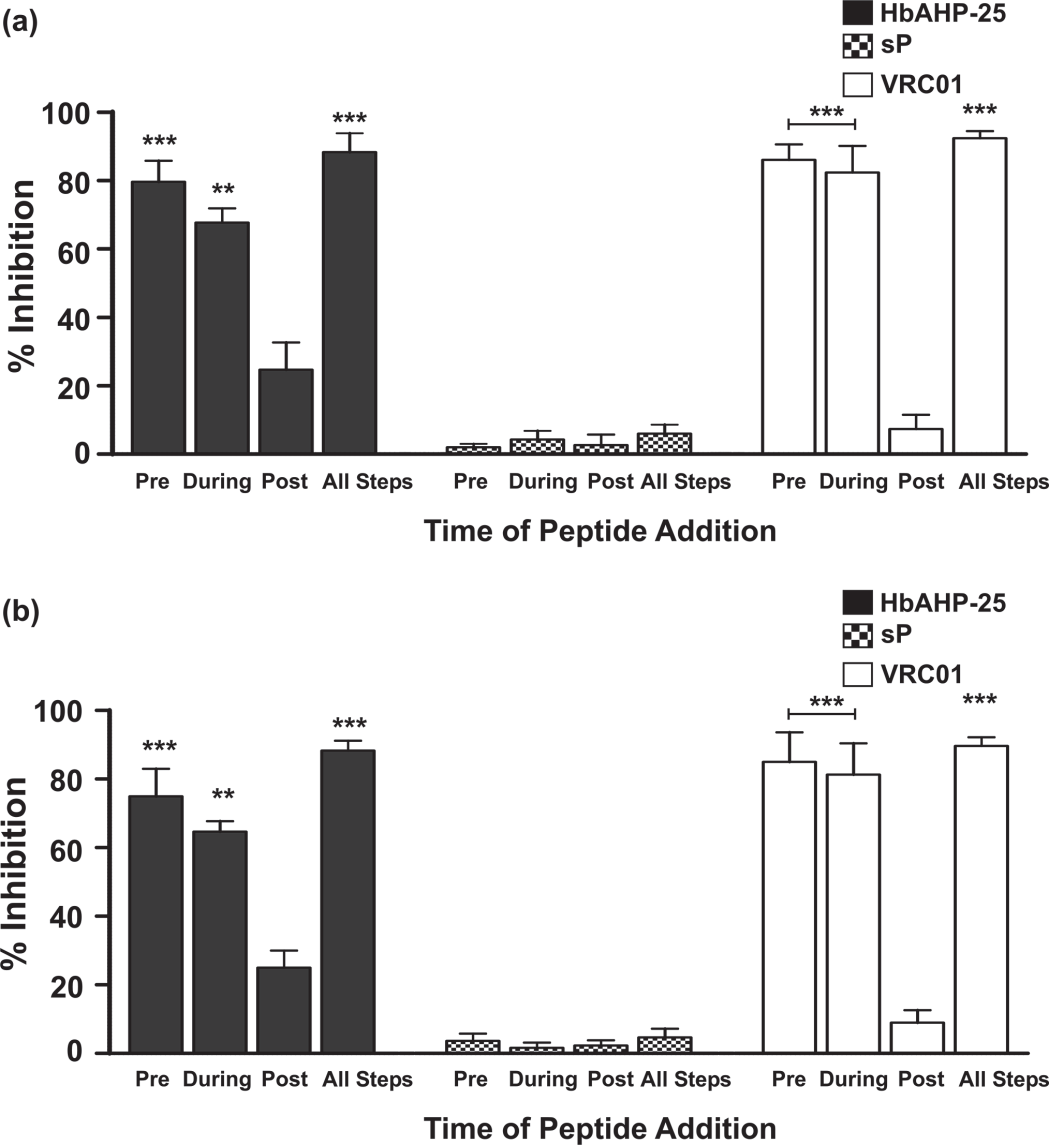
Effect of HbAHP-25 on different stages of HIV-1 infection. TZM-bl cells were infected with HIV-1 strains (IIIB) (a) and (Ada-5) (b) and HbAHP-25 was added during different stages of infection. (Pre: Viral strains pre-treated with the peptide were used for infecting TZM-bl cells; During: TZM-bl cells were simultaneously treated with viral strains and peptide; Post: TZM-bl cells were infected with HIV-1 virus first and 6 hrs later treated with the peptide and; all steps: HbAHP-25 was added at all the three steps. Luciferase activity was measured after 48 hrs of infection and per cent inhibition was calculated as mentioned earlier. sP and VRC01 were used as negative and positive controls respectively. Values are the mean (± SD) of six observations from three experiments performed on different days (**p<0.01; ***p<0.001).

Time-of-addition experiment was designed to gain an insight into the mechanism of anti-HIV-1 activity of the HbAHP-25. For this, the interaction time and order of HbAHP-25 addition was varied in four different ways. Results revealed that TZM-bl cells infected with HIV-1 IIIB and ADA5 strains, which were pre-incubated with HbAHP-25 (71.40 μM), showed significantly lesser luciferase activity compared with cells infected with HIV-1 IIIB/ADA5 not pre-incubated with HbAHP-25 (Fig 9). When HbAHP-25 and HIV-1 IIIB/ADA5 strains were simultaneously added to TZM-bl cells (peptide-virus mixture group), anti-HIV activity of the peptide was still significant. However, when TZM-bl cells were first infected with HIV-1 IIIB or HIV-1 ADA5 strains and then treated with HbAHP-25, anti-HIV activity of HbAHP-25 was significantly lesser than that shown by the first two groups. Maximum inhibition was observed when HbAHP-25 was present during all the three steps. Further, no inhibition was seen with scrambled peptide. Thus the anti-HIV activity of HbAHP-25 can be attributed to its direct interaction with HIV-1.

### HbAHP-25 has specificity for gp120 of HIV-1

**Fig 10:**
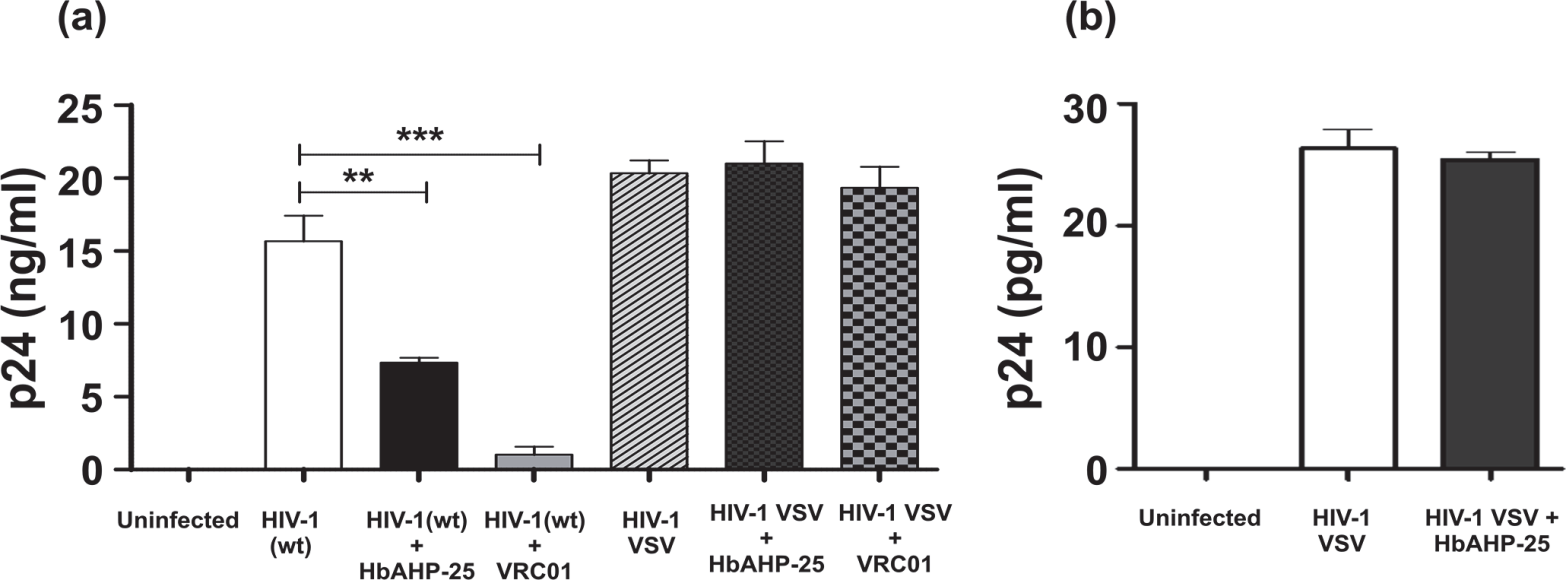
HIV-1 VSV infection in the presence of HbAHP-25. a) CEM-GFP cells were infected for 4 hrs with HIV-1 (wt) and HIV-1 VSV pre-incubated with 35.7 μM of HbAHP-25. p24 levels were checked on day 4 post infection. VRC01 (0.1μM) was used as a control. b) Intracellular p24 levels in CEM-GFP cells, infected for 1 hr with HIV-1 VSV pre-incubated with HbAHP-25 (1 hr). Values are the mean (± SD) of three different biological replicates performed on different days (**p<0.01; ***p<0.001).

To determine the specificity of HbAHP-25 interaction with gp120, HIV-1 (R9∆E) was pseudotyped with envelope protein from vesicular stomatitis virus (VSV-G) and p24 levels were measured as mentioned in S4 Fig. Equal amounts (20ng/ml p24) of HIV-1 (wt) and HIV-1 VSV were separately incubated with 35.7 μM HbAHP-25 for 1 hr. As shown in Fig 10A, infectivity of HIV-1 VSV was not affected by its pre-incubation with HbAHP-25. HIV-1 VSV was able to infect and replicate in these cells, as indicated by p24 levels after day 4 of infection. In contrast, as shown above, HIV-1 (wt) infectivity was inhibited by its pre-incubation with HbAHP-25. Further, pseudotyped HIV-1 was able to enter the cells within one hour even in the presence of HbAHP-25, as represented by intracellular p24 levels (Fig 10B). This clearly indicated that HbAHP-25 doesn’t interfere with the entry of HIV-1 VSV through endocytosis [39, 40] and anti-HIV activity of HbAHP-25 specifically relies on its interaction with gp120 of HIV-1.

### Seminal plasma and vaginal fluid does not affect anti-HIV activity of HbAHP-25

**Fig 11:**
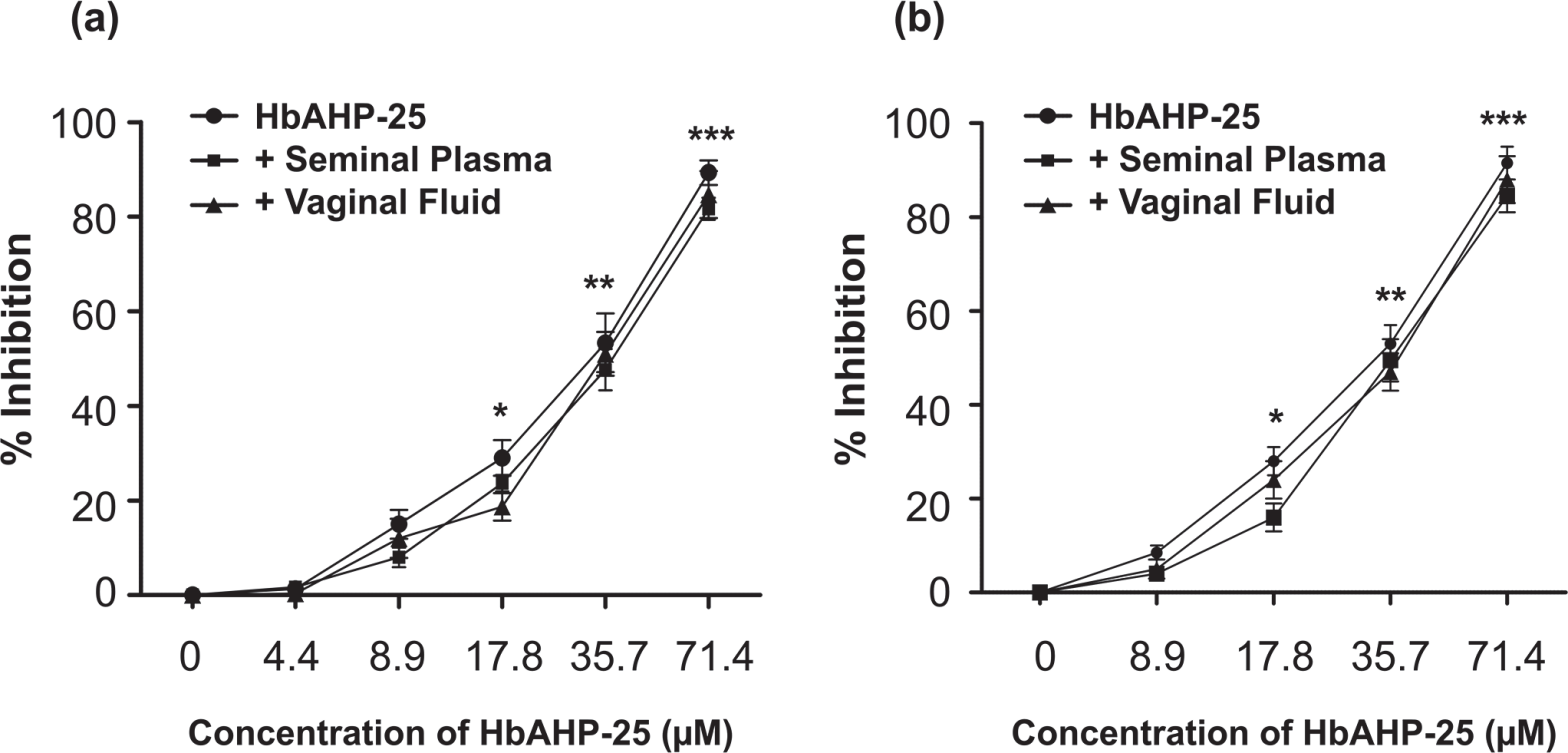
Anti-HIV activity of HbAHP-25 in the presence of seminal plasma and vaginal lavage. Luciferase activity in TZM-bl cells infected with HIV-1 IIIB (a) or HIV-1 Ada (b) in the presence of seminal plasma/vaginal lavage mixed with various concentrations of HbAHP-25. Anti-HIV activity imparted by vaginal fluid and seminal plasma was subtracted from the HbAHP-25 treated wells. (Control: Inhibition of HIV-1 by HbAHP-25 in absence of seminal plasma and vaginal fluid; +Seminal Plasma: Anti-HIV activity of HbAHP-25 in presence of seminal fluid; and +Vaginal Lavage: Anti-HIV activity of HbAHP-25 in presence of vaginal lavage) (*p<0.05; **p<0.01; ***p<0.001).

Next, we investigated whether HbAHP-25 retains its anti-HIV activity in biological fluids such as seminal plasma and vaginal fluid. Seminal plasma and vaginal fluid did not alter the anti-HIV potency of HbAHP-25 as we did not observe any significant change in the anti-HIV activity of HbAHP-25 in the presence of these fluids (Fig 11A). Seminal plasma and vaginal fluid showed some inherent anti-HIV activity, consistent with some previous reports [35, 37], which was subtracted from the cells infected with HIV-1 strains pre-incubated with HbAHP-25. Similar results were obtained for HIV-1 ADA5 strain (Fig 11B).

**Fig 12:**
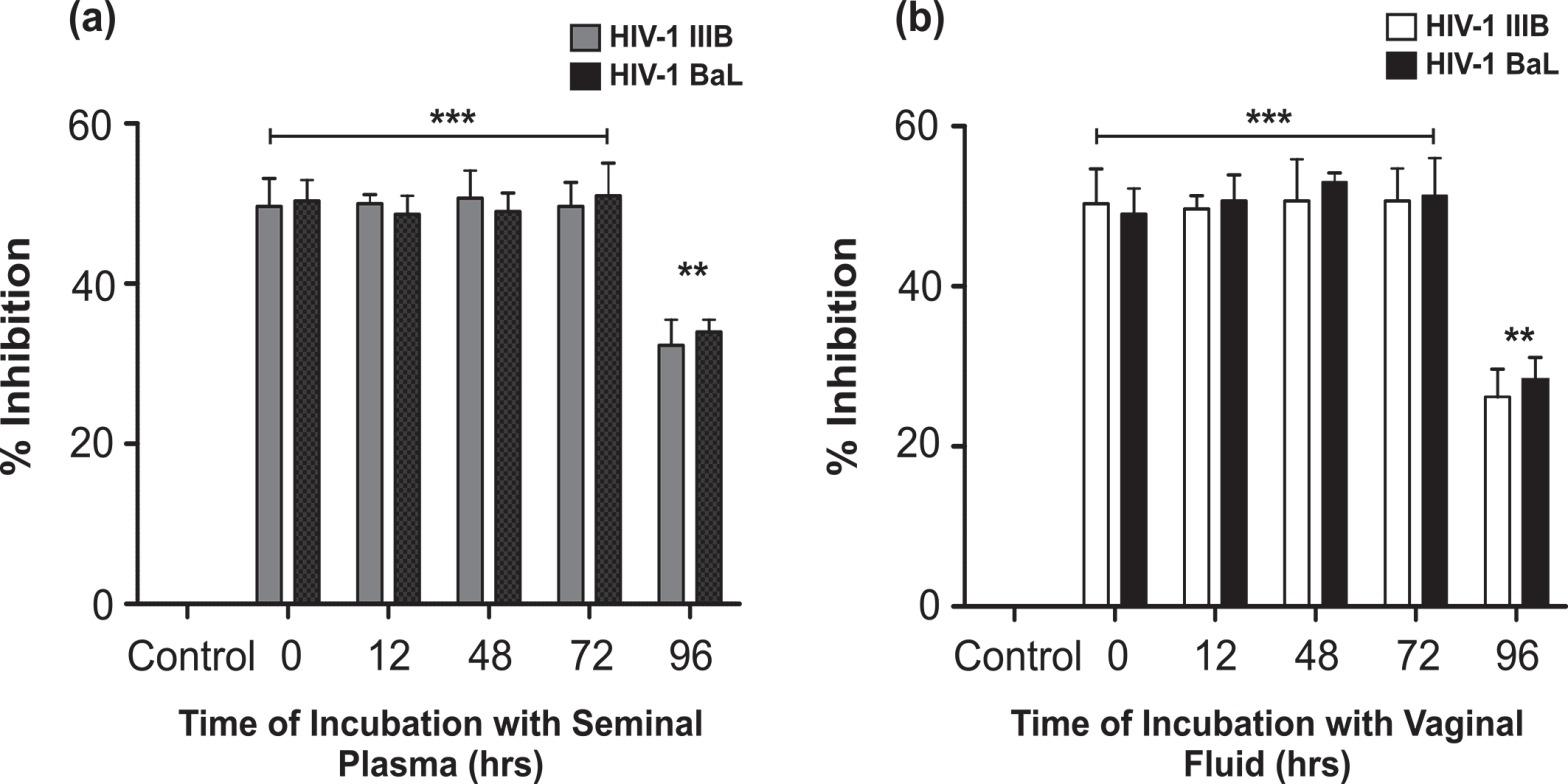
Anti-HIV activity of HbAHP-25 at different time points of incubation with seminal plasma/vaginal fluid. TZM-bl cells infected with either HIV-1 IIIB or HIV-1 BaL in the presence of HbAHP-25 (pre-incubated with seminal plasma [a] and vaginal fluid [b] for indicated time points). Luciferase activity was measured after 48 hrs of infection and % inhibition was calculated. Values are the mean (± SD) of three different biological replicates performed on different days (**p<0.01; ***p<0.001).

Stability of HbAHP-25 in seminal plasma and vaginal fluid was evaluated by incubating the peptide (35.7 μM) with seminal plasma and vaginal fluid for different time periods. We did not observe any significant loss in anti-HIV activity of HbAHP-25 when pre-incubated with seminal plasma and vaginal fluid for up to 72 hrs (Fig 12). These findings suggest that HbAHP-25 has good stability, ideal for development of a microbicide.

### HbAHP-25 displays α-helical conformation

**Fig 13:**
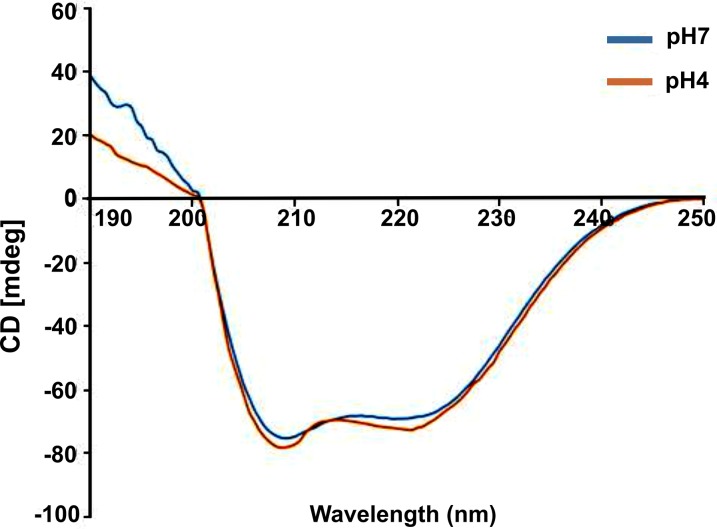
Structural conformation of HbAHP-25 at pH 7 and pH 4. 10μM of HbAHP-25 dissolved in PBS and sodium acetate buffer was acquired in a cuvette with 0.1cm path length, and 5.0nm bandwidth. The figure represents CD spectra of one of the accumulated scans of HbAHP-25, analyzed on CD spectrometer at 37°C.

Further the structure of HbAHP-25 at pH4 and pH7 was analyzed using CD spectroscopy. HbAHP-25 showed mostly α-helical structure, characterized by a negative peak in the far UV region of CD spectrum (Fig 13). The α-helical content of HbAHP-25 was found to be about 50%. In addition to this, there was no significant change in its structure at low pH. This indicates that HbAHP-25 retains its structure at different pH, essential for retaining its anti-HIV activity in different conditions.

## Discussion

In the past two decades, several natural antimicrobial peptides (AMPs) have been identified. Some of these AMPs such as defensins, human neutrophil peptides (HNPs), indolicidin, secretory leucocyte protease inhibitor (SLPI), magainins etc, are reported to have anti-HIV activity [8–14, 41]. Unexpectedly, AMPs like human defensin-5 and 6 have been shown to exacerbate HIV infection by concentrating virus to the target cells [42]. Moreover, some of the AMPs cause adverse effects on vaginal commensals like lactobacilli which are required to maintain low pH in vaginal mucosa [43]. Considering these limitations and unavailability of an effective prophylactic HIV vaccine, there is a need to identify novel peptides/molecules which can prevent HIV-1 infection at initial stages and counter the sabotage of host immune system by HIV.

Hemoglobin (Hb), conventionally known for its oxygen carrying capacity, has been shown to have antimicrobial activity [44]. In the recent years, data have emerged to suggest the role of Hb and its peptides in protection against microbial infection [15–17, 45]. Hemocidins (Hb-derived peptides) with antimicrobial activity have been detected in human uterine fluid [46], vaginal lavage [47] and menstrual blood [18]. Hemocidins play an essential role in maintaining vaginal immunity by exhibiting antibacterial activity and alleviating inflammation caused by bacteria. They potentiate the activity of various known antimicrobials present in the female genital tract. It is also noteworthy that hemocidins are active in different ionic strengths and also at low pH, characteristic of female genital tract. Hemoglobin and Hb derived peptides have been shown to play essential protective role during menstruation [18, 19]. Hence, we explored whether Hb-derived peptides have anti-HIV activity.

To begin with, *in-silico* methods were used to determine whether Hb-α subunit and its peptides have ability to bind with CD4 binding domain of gp120 protein of HIV-1. CD4 binding site of gp120 is highly conserved and functionally important for entry/fusion of HIV into the host cells. Thus CD4 binding domain of gp120 appeared to be a good target for intervention. However, the existing structure database lacked the entry for unliganded glycosylated gp120 of HIV-1 during the initial stage of our study. Hence, we used 2BF1 (unliganded and glycosylated SIV gp120) as the template to model the structure of gp120. *In-silico* studies identified 3 Hb- subunit derived peptides which interact with CD4 binding domain of gp120 of HIV-1. An analogue of one of these peptides, named as HbAHP-25 (hemoglobin derived anti-HIV peptide) showed maximal binding to CD4 binding site of gp120. HbAHP-25 interacts with E13, V38, G39, K129, R281, R285 and K290 amino acids of gp120. These residues fall within or proximal to CD4 binding site of gp120. Comparison of the amino-acid residues of gp120 actively interacting with HbAHP-25/CD4/X5 antibody revealed that HbAHP-25 binds proximal to CD4 binding site of gp120 other than that recognized by X5 antibody.

HbAHP-25 binds to CD4 binding pocket on gp120 with high affinity and blocks interaction of gp120 with CD4. Further, HbAHP-25 exerts its inhibitory activity, irrespective of the tropism of HIV-1. HbAHP-25 showed activity against both CXCR4 and CCR5 tropic viral strains. This is indeed a significant observation considering that majority of the HIV entry inhibitors are either CCR5 antagonists (e.g. Cencriviroc) [48] or CXCR4 antagonists (e.g. AMD3100) [49]. Enfuvirtide, another anti-HIV peptide, has been shown to interfere with the HIV-host cell fusion. However, long term treatment with Enfuviritide leads to resistance due to mutations in the drug interacting region of gp41 [50]. We presume that HbAHP-25 treatment will not induce resistance as HbAHP-25 interacts with some key CD4 interacting residues in gp120 and mutations in these residues would be detrimental to virus infection.

Although HbAHP-25 exerts its anti-HIV effect at high concentration, its activity is highly specific. Its activity is directed more towards the virus than to host cells. Some of the known anti-HIV peptides such as defensins have better selectivity index, but many of such peptides/molecules act as a double edged-sword. These peptides inhibit HIV infection but also lead to inflammation and/or potentiate pathogen invasion [51]. Our study revealed that HbAHP-25 does not bind to any of the cell surface proteins, indicating the specificity of its binding to viral gp120 or to the cells expressing gp120. Further, HbAHP-25 was not inhibitory to lactobacilli (data not shown), essential for maintaining vaginal homeostasis. Our preliminary analysis reveals that HbAHP-25 does not cause disruptions of tight junctions and also does not induce any inflammation in vaginal/endocervical epithelial cells (Tahir *et al*, unpublished). It indirectly hints at the safety of HbAHP-25. Our previous study also reported that RVFHbαP, another hemoglobin derived peptide does not have inflammatory effects on vaginal epithelial cells [32]. This is important in view of the fact Nonoxynol-9 failed in clinical trials as it caused detrimental effects on vaginal mucosa [52].

It is essential for an ideal microbicide to retain its activity at low pH. Our structural studies indicated that HbAHP-25 maintains its structure at neutral as well as acidic pH. In addition, HbAHP-25 retains its anti-HIV potency in the presence of biological fluids, such as semen and vaginal fluid. However, it is important to evaluate anti-HIV efficay of this peptide against various clinical isolates as well. Notwithstanding this limitation, the current study may still make a significant contribution towards the development of safe and effective anti-HIV agents.

In conclusion, the study shows that HbAHP-25, a molecule derived from human hemoglobin, has significant anti-HIV activity. It inhibits HIV at the initial steps of infection to the target cells. Further, it is non-cytotoxic to host cells and is active in broad range of pH. It would be interesting to analyze whether the anti-HIV activity of HbAHP-25 is potentiated when used in combination with other anti-HIV agents. It is also essential to demonstrate *in vitro* and *in vivo* efficacy of HbAHP-25 against clinical isolates of HIV and also its safety before its clinical use. Studies are in progress to evaluate the efficacy of HbAHP-25 *in vivo*.

## Supporting Information

S1 Fig Binding of designed peptides to gp120. Peptides that were designed *in silico* were evaluated for their binding to gp120 of HIV-1 IIIB. Peptides were coated on 96 well microtiter plate for 16 hrs and then incubated with 500ng of gp120 for 2 hrs at 37°C. This was followed by addition of anti-gp120 antibody, and absorbance was read at 492nm. Background absorbance was subtracted from the wells where peptides were not coated. Peptide 1 and peptide 3 bind to gp120 while peptide 2 failed to show any binding (*p<0.05; **p<0.01; ***p<0.001).
(TIF)

S2 Fig Anti-HIV activity of 3 designed peptides. Anti HIV activity of three peptides was determined using H9 cells. H9 cells were infected for 4 hrs with 0.1 MoI of HIV-1 IIIB pre-incubated with various concentrations of peptides. Peptides were added post infection as well. Supernatant was collected on day 4 post infection, and p24 antigen assay was performed. Peptide 1 and 3 showed anti-HIV activity, whereas peptide 2 failed to demonstrate any activity (**p<0.01; ***p<0.001).
(TIF)

S3 Fig Anti-HIV activity of peptide analogues of peptide 1. After modifying the peptide 1, we performed anti-HIV assay as earlier. HIV-1 IIIB was pre-incubated with peptide analogues for 1 hr and then added to H9 cells for 4 hrs. Levels of p24 levels were determined on day 4 post infection. Anti-HIV activity of peptide analogue-1b was significantly enhanced (*p<0.05; **p<0.01; ***p<0.001).
(TIF)

S4 Fig p24 levels of HIV-1 (wt) and HIV-1 VSV virus. CEM-GFP cells were infected with both viruses for 4 hrs, washed and collected supernatants at day 1, 2, & 3. p24 levels were then measured.
(TIF)

S1 Table Binding energies and residual interactions of peptides with gp120. This table represents the binding energies of each peptide with liganded and unliganded gp120 and amino acids from gp120 interacting with the respective peptides. The numbers in the bracket shows their actual number in the crystal structure (gp120) while the number preceding them is the renumbered residual position during homology modelling. All these peptides taken for docking showed good interactions with the gp120, however peptides 1 and its analogues 1b, and peptide-3 have better binding energies than peptide analogue 1a and peptide-2.
(DOCX)
