# Supplementary figures and images for: HbAHP-25, an In-Silico Designed Peptide, Inhibits HIV-1 Entry by Blocking gp120 Binding to CD4 Receptor
Source: PLoS One. 2015 Apr 27;10(4):e0124839. doi: 10.1371/journal.pone.0124839 (PMC4411102; doi:10.1371/journal.pone.0124839)

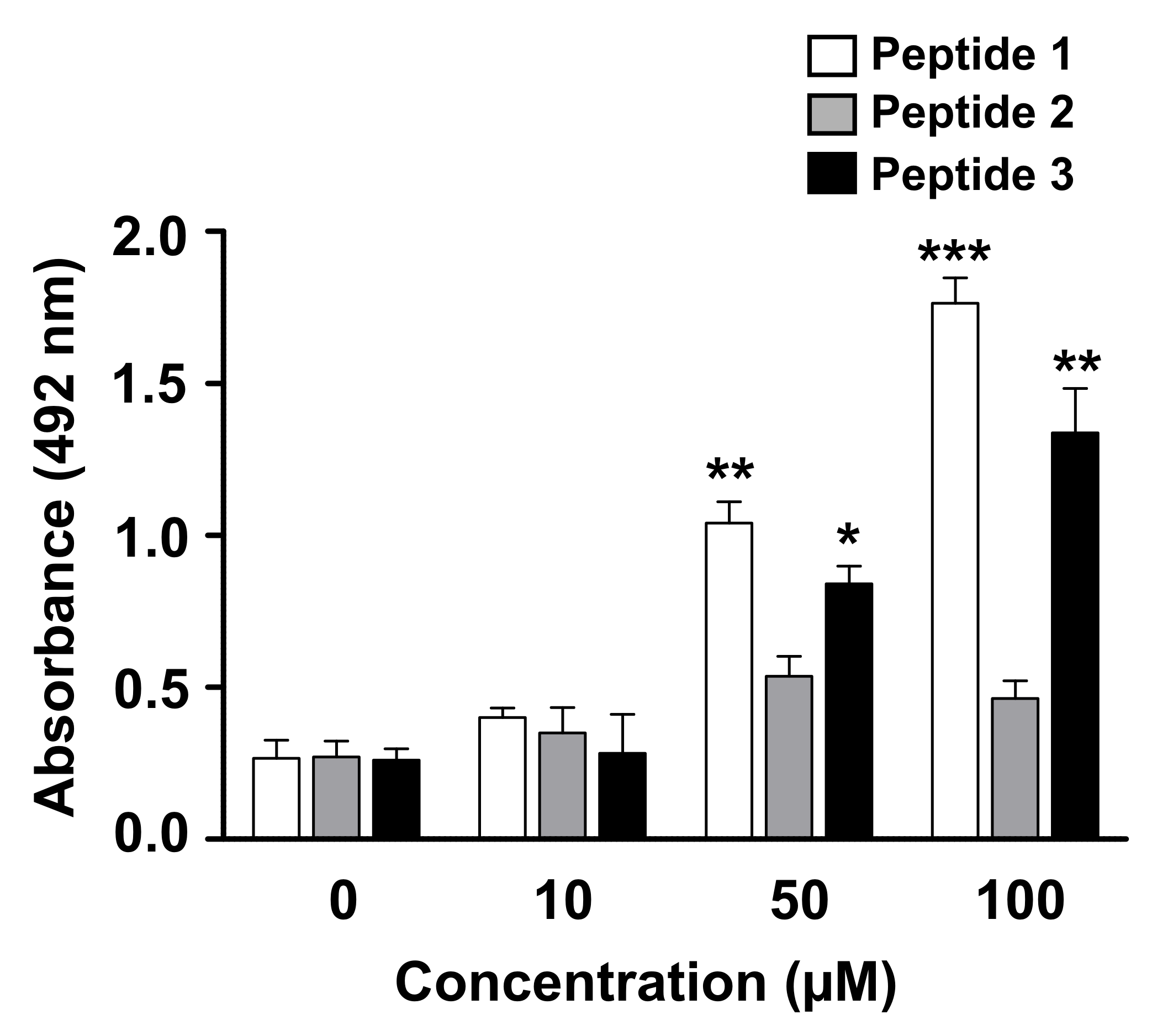

Supplement: S1 Fig — Peptides that were designed in silico were evaluated for their binding to gp120 of HIV-1 IIIB. Peptides were coated on 96 well microtiter plate for 16 hrs and then incubated with 500ng of gp120 for 2 hrs at 37°C. This was followed by addition of anti-gp120 antibody, and absorbance was read at 492nm. Background absorbance was subtracted from the wells where peptides were not coated. Peptide 1 and peptide 3 bind to gp120 while peptide 2 failed to show any binding (*p<0.05; **p<0.01; ***p<0.001). (TIF) [file pone.0124839.s001.tif]

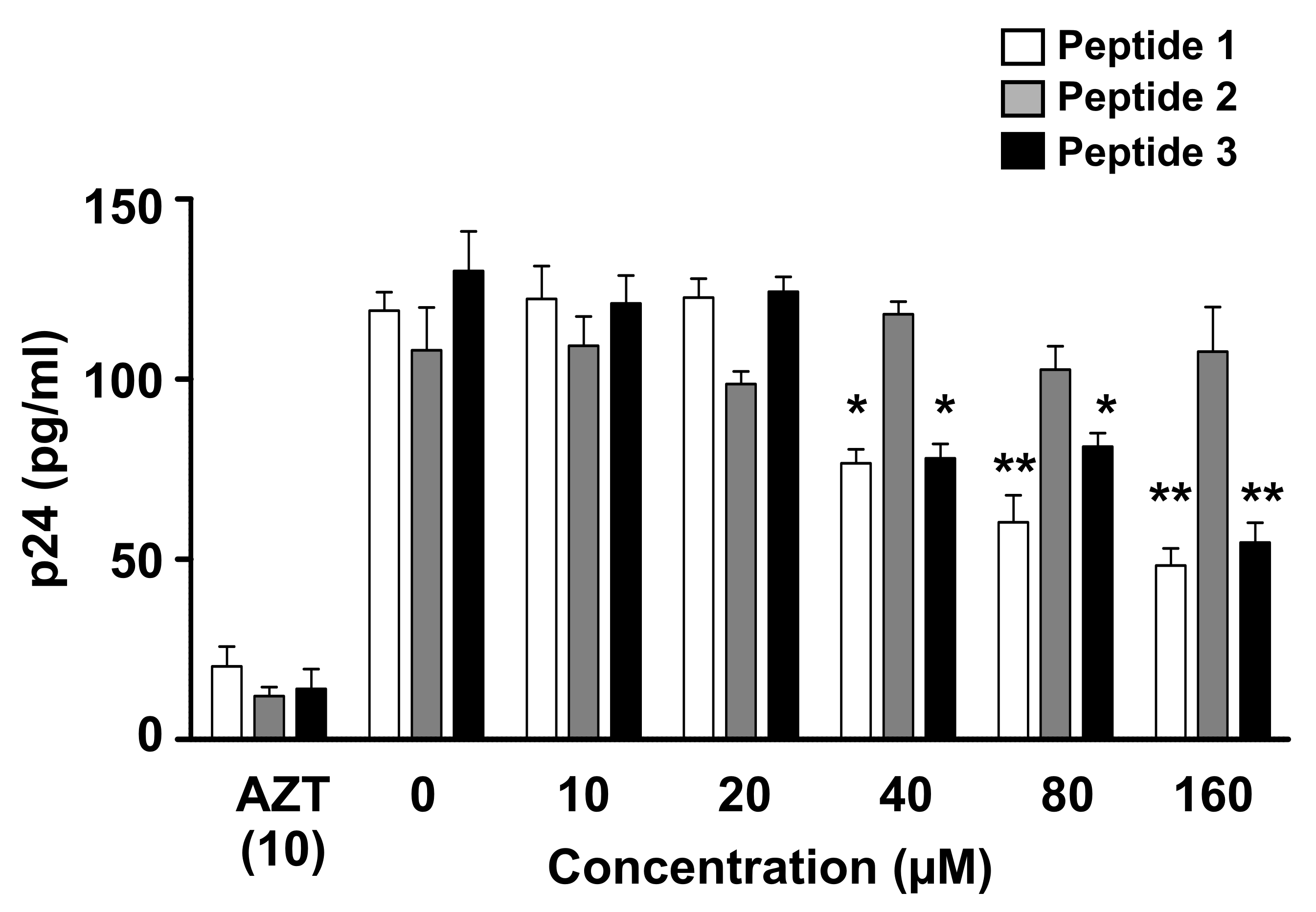

Supplement: S2 Fig — Anti HIV activity of three peptides was determined using H9 cells. H9 cells were infected for 4 hrs with 0.1 MoI of HIV-1 IIIB pre-incubated with various concentrations of peptides. Peptides were added post infection as well. Supernatant was collected on day 4 post infection, and p24 antigen assay was performed. Peptide 1 and 3 showed anti-HIV activity, whereas peptide 2 failed to demonstrate any activity (**p<0.01; ***p<0.001). (TIF) [file pone.0124839.s002.tif]

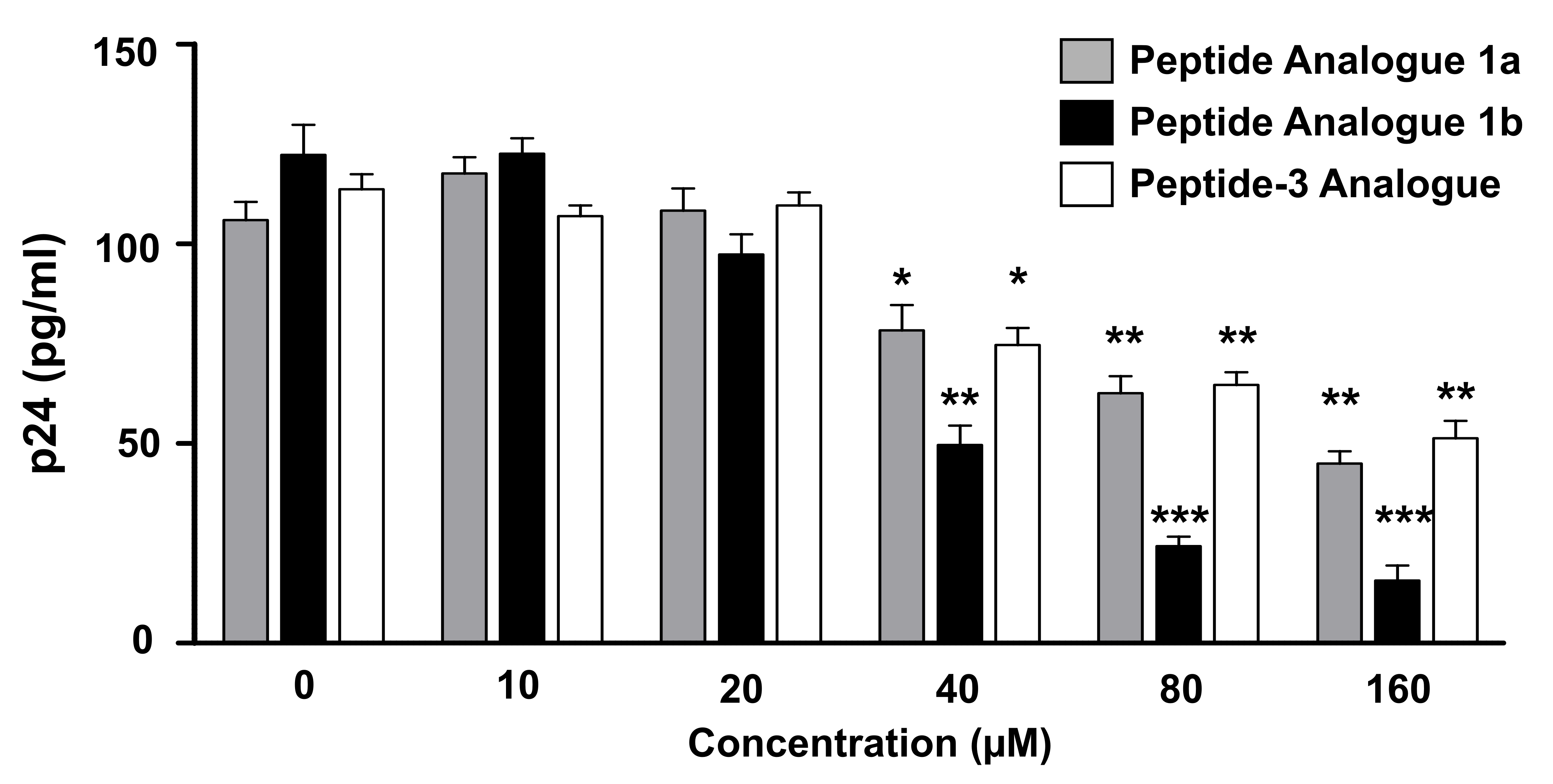

Supplement: S3 Fig — After modifying the peptide 1, we performed anti-HIV assay as earlier. HIV-1 IIIB was pre-incubated with peptide analogues for 1 hr and then added to H9 cells for 4 hrs. Levels of p24 levels were determined on day 4 post infection. Anti-HIV activity of peptide analogue-1b was significantly enhanced (*p<0.05; **p<0.01; ***p<0.001). (TIF) [file pone.0124839.s003.tif]

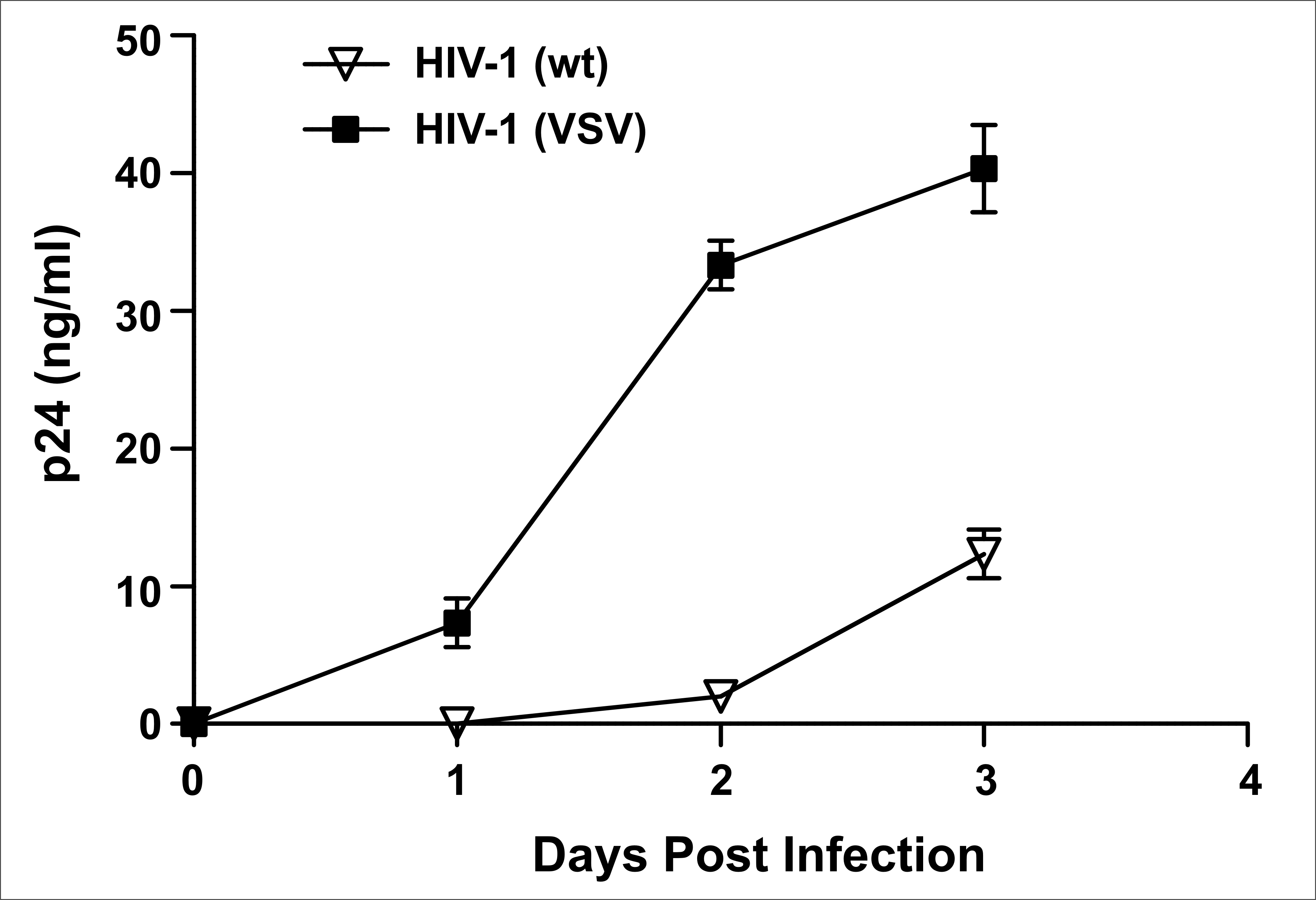

Supplement: S4 Fig — CEM-GFP cells were infected with both viruses for 4 hrs, washed and collected supernatants at day 1, 2, & 3. p24 levels were then measured. (TIF) [file pone.0124839.s004.tif]
