# Supplementary material for: HbAHP-25, an In-Silico Designed Peptide, Inhibits HIV-1 Entry by Blocking gp120 Binding to CD4 Receptor
Source: PLoS One. 2015 Apr 27;10(4):e0124839. doi: 10.1371/journal.pone.0124839 (PMC4411102; doi:10.1371/journal.pone.0124839)
Supplement: S1 Table — This table represents the binding energies of each peptide with liganded and unliganded gp120 and amino acids from gp120 interacting with the respective peptides. The numbers in the bracket shows their actual number in the crystal structure (gp120) while the number preceding them is the renumbered residual position during homology modelling. All these peptides taken for docking showed good interactions with the gp120, however peptides 1 and its analogues 1b, and peptide-3 have better binding energies than peptide analogue 1a and peptide-2. (DOCX) [file pone.0124839.s005.docx]

| **Peptides** | **Cluster size** | **Binding energy of peptides with unliganded gp120 (kcal/mol)** | **Binding energy of peptides with liganded gp120 (kcal/mol)** | **Residual interactions of peptides with unliganded gp120** |  |
| --- | --- | --- | --- | --- | --- |
| Peptide-1 | 251 | -14.52 | -9.42 | T1(90), E13(102), G39(128), K129(282), R281(476), R285(480), K290(485), Y291(486) |  |
| Peptide analogue- 1a | 143 | -13 | -12.4 | V89(242),S90(243),Q93(247),  C94(248) |  |
| Peptide-analogue1b | 168 | -16.64 | 12.1 | C86(239),T87(240),T130(283),S182  (364),K290(485) |  |
| Peptide-2 | 111 | -10.08 | -9.9 | D10(99),E17(106),C43(196),  T45(198) |  |
| Peptide-3 | 212 | -14.12 | -10.5 | D24(113),I98(251) |  |

**Supporting Table**
